# Supplementary material for: Exposure to air pollution and scarlet fever resurgence in China: a six-year surveillance study
Source: Nat Commun. 2020 Aug 25;11:4229. doi: 10.1038/s41467-020-17987-8 (PMC7447791; doi:10.1038/s41467-020-17987-8)
Supplement: Supplementary file 1 — Supplementary Information [file 41467_2020_17987_MOESM1_ESM.pdf]

Supplementary Information

Exposure to air pollution and scarlet fever resurgence in China: a six-year surveillance study

Yonghong, et.al

Contents

Supplementary Figures 1
Supplementary Figure 1 1
Supplementary Figure 2 2
Supplementary Figure 3 3
Supplementary Figure 4 4
Supplementary Figure 5 5
Supplementary Figure 6 6
Supplementary Figure 7 7
Supplementary Figure 8 8
Supplementary Figure 9 9
Supplementary Figure 10 10
Supplementary Figure 11 11
Supplementary Figure 12 12
Supplementary Figure 13 13
Supplementary Tables 14

Supplementary Table 1 14
Supplementary Table 2 15
Supplementary Table 3 16
Supplementary Table 4 17

Supplementary Figure 1. Seasonal pattern of scarlet fever notifications during pre-upsurge period (2004-2011) and post-upsurge period (2011-2018), based on the monthly incidence rate per 100 000 population in all of China

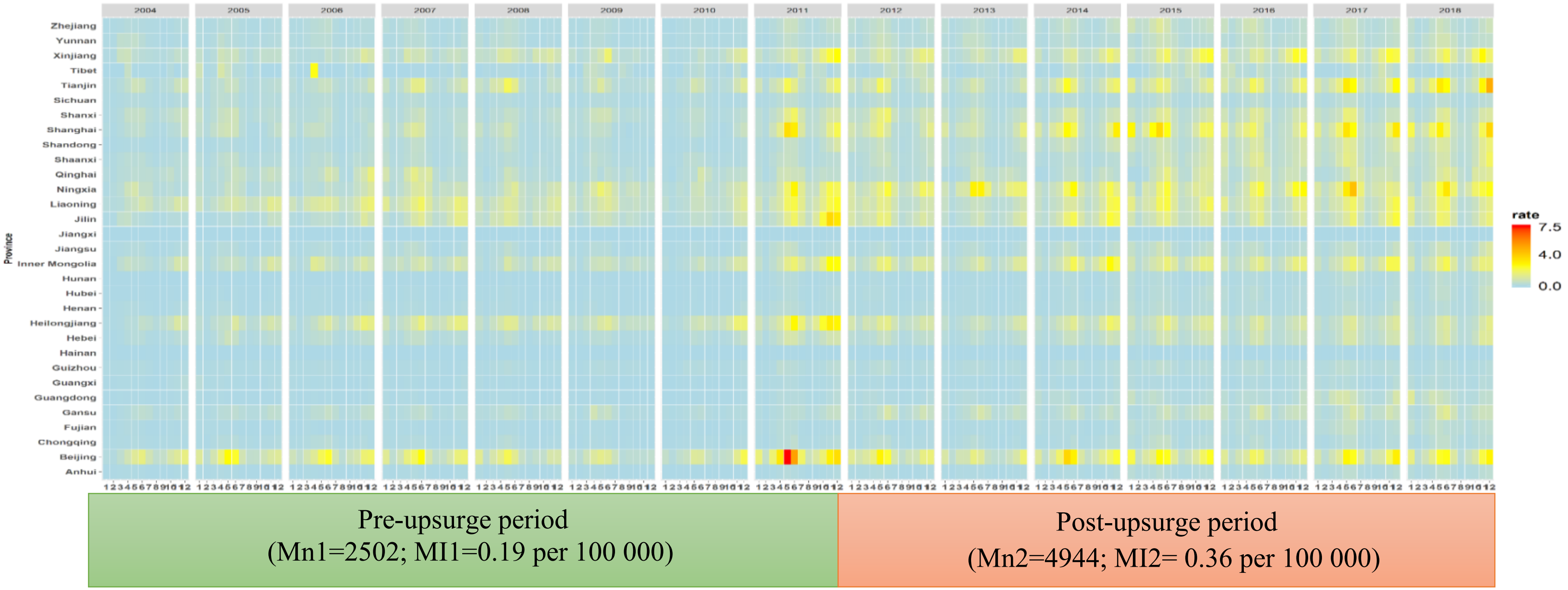

Notes: Mn1= average monthly number of cases 2004-10; Mn2= average monthly number of cases 2011-18

MI1= average monthly incidence 2004-10 (per 100,000); MI2= average monthly incidence 2011-18 (per 100,000).

Description: The seasonal patterns were similar during these two periods, with semiannual peaks of activity, including a major peak in May and June followed by a smaller peak in November and December. The subsequent seasons saw a return of increased scarlet fever activity during 2011-2018.

Supplementary Figure 2. Spatiotemporal distribution of scarlet fever cases in China, 2004-18

a.

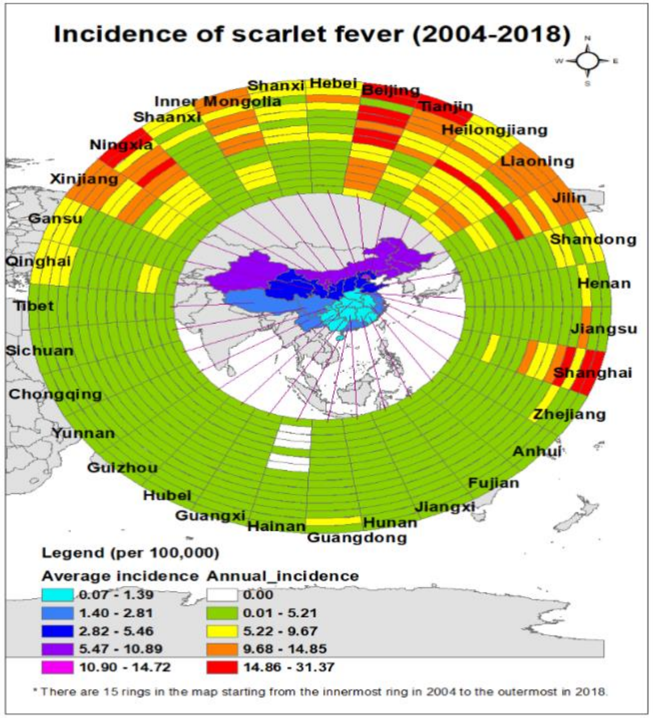

b.

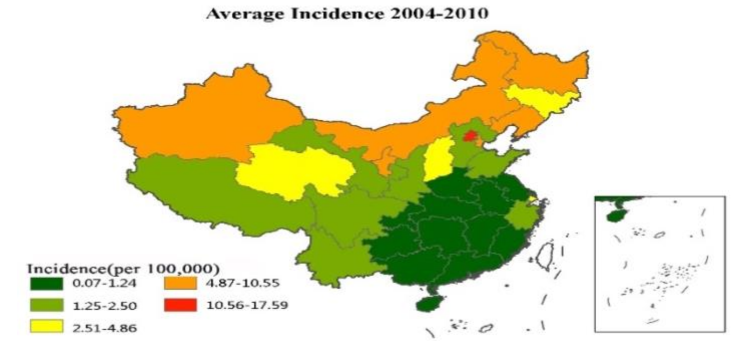

c.

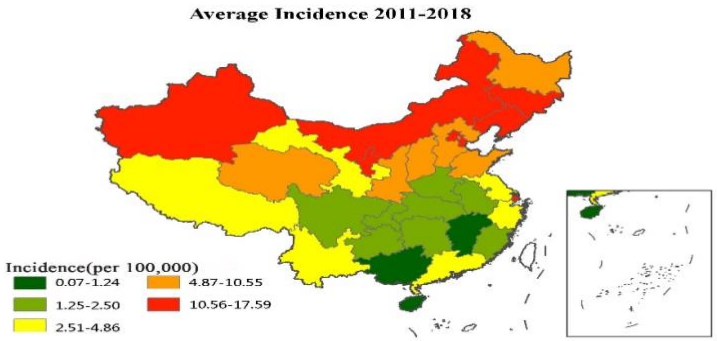

Notes: a. Annual incidence of scarlet fever per 100,000 people in the 31 Chinese provinces investigated. The 13 rings contain data for each year studied, with the innermost ring bearing data for 2004, and moving outwards through the years to the outermost ring bearing data for 2018. b. Choropleth maps of the average annual incidence of scarlet fever, by region, based on the annual incidence per 100,000 people in China in 2004-10, before the upsurge in the incidence of infections. c. Choropleth maps of the average annual incidence of scarlet fever, by region, based on the annual incidence per 100,000 people in China in 2011-18, after the upsurge in the incidence of infections. Description: Scarlet fever was predominantly distributed in the north, northeast, and northwest of China. All parts of China had marked increases in the incidence of scarlet fever during 2011-2018. The provinces with a latitude higher than 33.4 degrees north had a higher annual incidence than those at lower latitudes.

Supplementary Figure 3. Spatiotemporal distribution of six air pollutants in China, 2013-2018

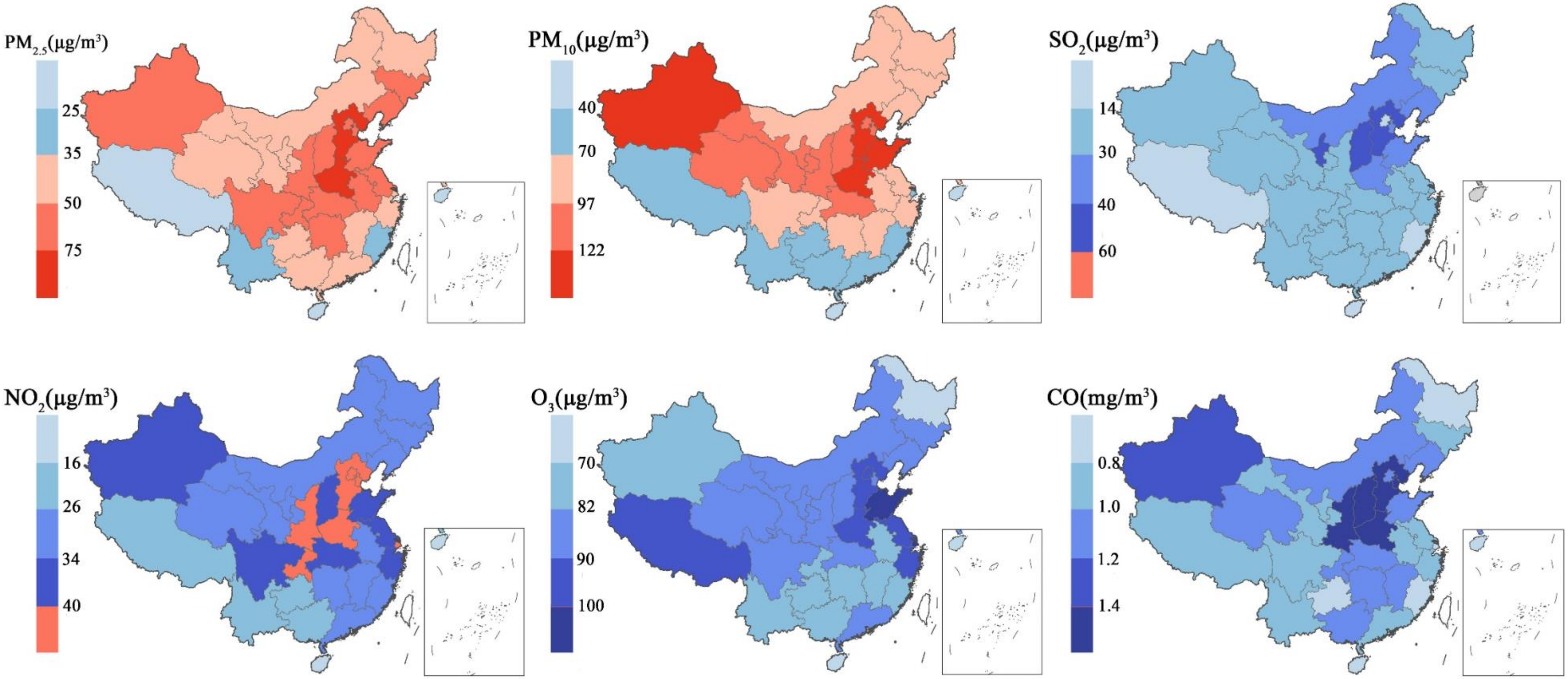

Notes: Choropleth maps of the average annual value of air pollutants by region, based on the annual value during 2013-2018. Red means over China guidelines II (issued in 2018); Blue: means below China guidelines II. Depth of color denotes the air pollutants' concentration.

Supplementary Figure 4. Time series plot of the monthly mean air pollution concentrations at high and low degrees of latitude of China, 2013-2018

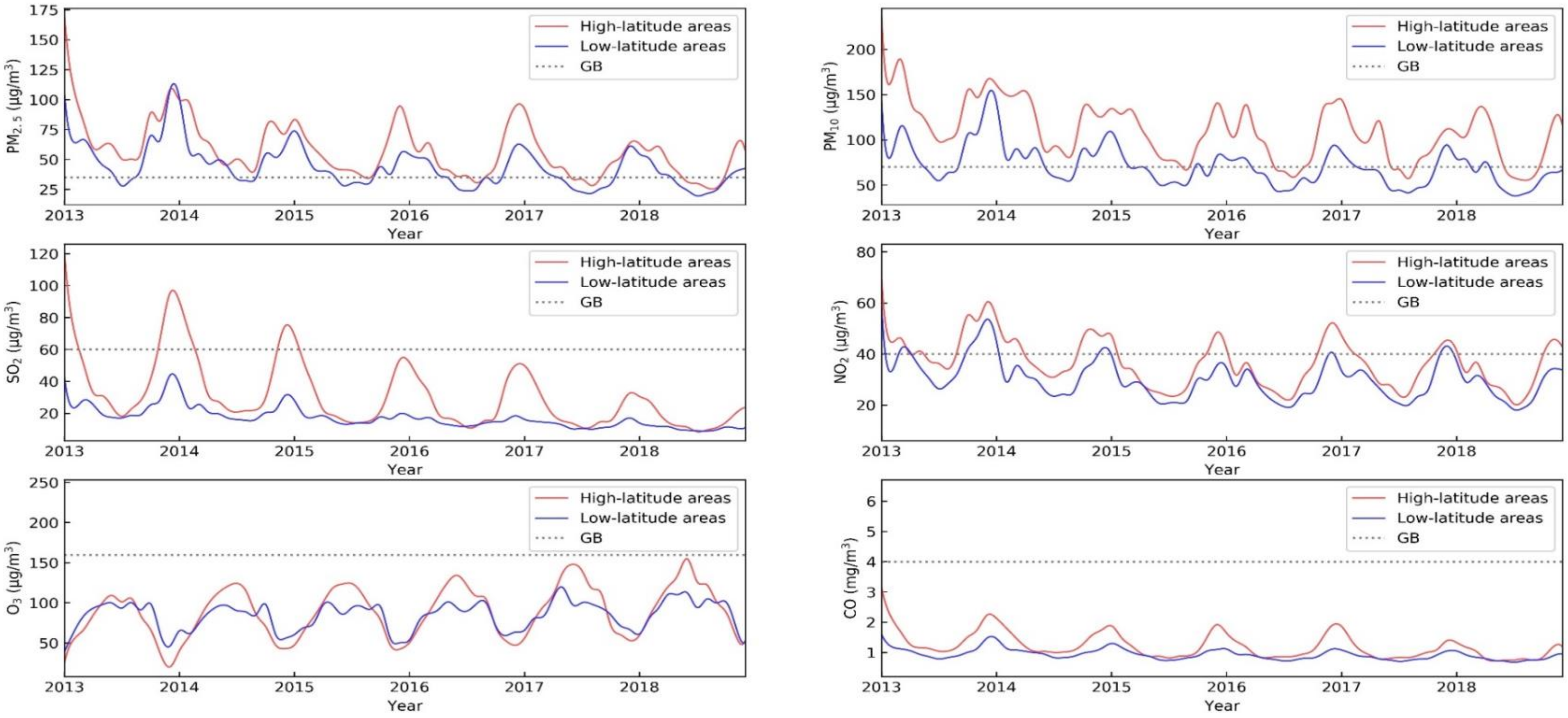

Notes: $PM_{2.5}$=particulate matter with aerodynamic diameter less than 2.5μm. PM_{10}=particulate matter with aerodynamic diameter less than 10μm. NO_2=nitrogen dioxide. SO_2=sulfur dioxide. O_3=ozone. GB= National Standard of the People's Republic of China (Issued in 2018)

Description: The concentrations of air pollutants peaked in December to February in both high- (≥33.4 northern degrees) and low-latitude areas (<33.4 northern degrees).

Supplementary Figure 5. The boxplot of six air pollutants in four quantile regions of average scarlet fever incidences from 2013 to 2018

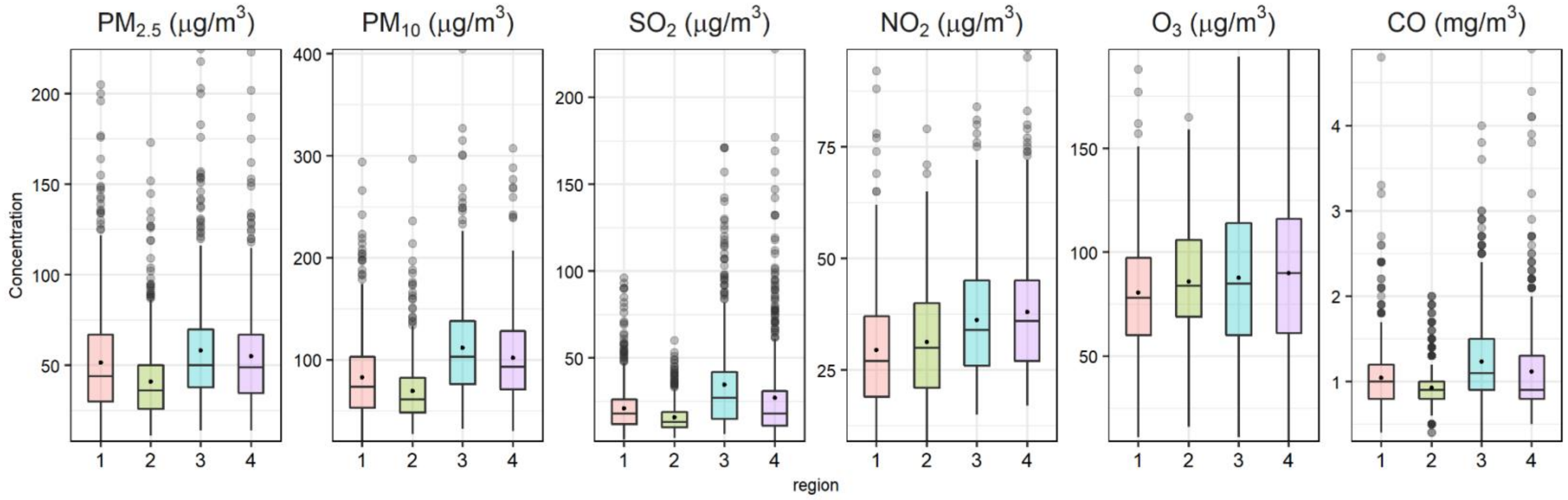

Notes: The average incidences in 31 provinces of China are divided into four quantile regions from 2013 to 2018. The cut-off points of average incidences for the first, second, third and fourth groups are <=25th percentile, > 25th percentile and <=50th percentile, >50th percentile and <=75 percentile, and >75 percentile.

Supplementary Figure 6. Box-plots of six weather variables before and after 2011 in China

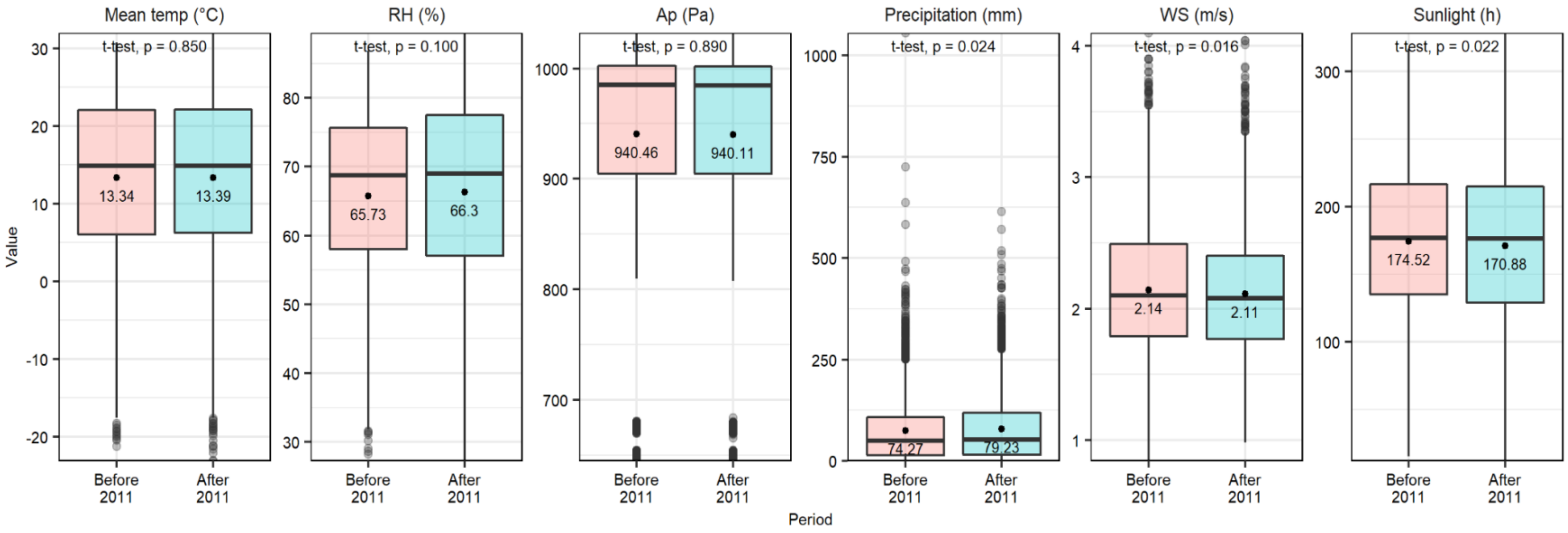

Notes: There are three significantly different variables ($p<0.05$) including precipitation, wind speed, and sunlight hours.

RH= Relative humidity; Ap=Air pressure; WS=Wind speed.

Supplementary Figure 7. The boxplots of six meteorological conditions and the number of scarlet fever cases in four seasons from 2004 to 2018

a. Six meteorological conditions

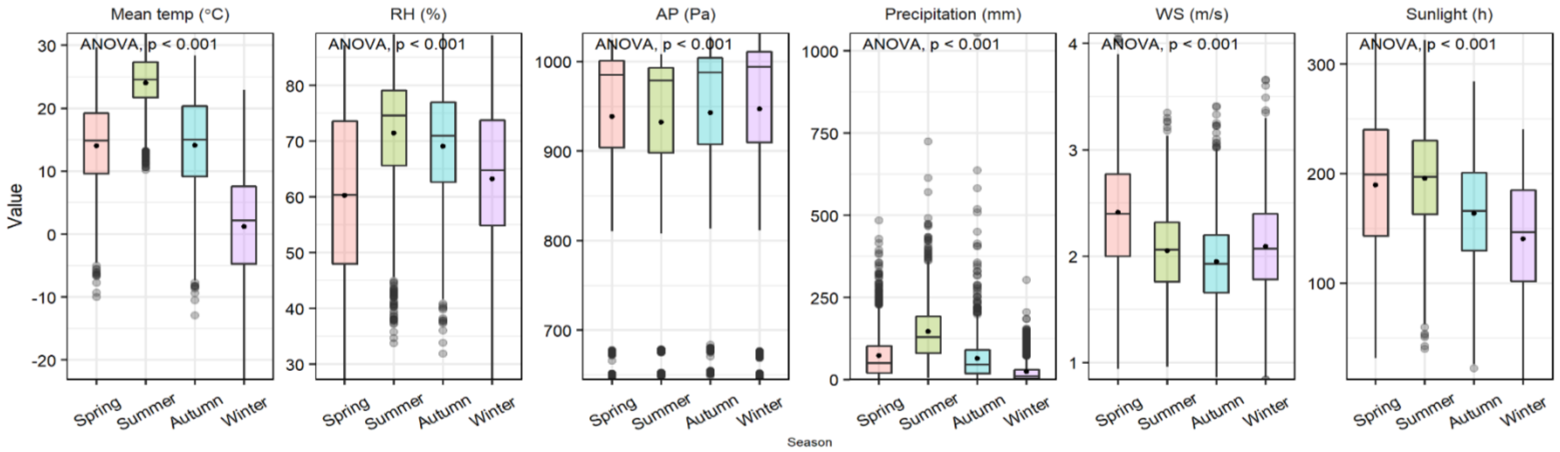

b. Number of scarlet fever cases

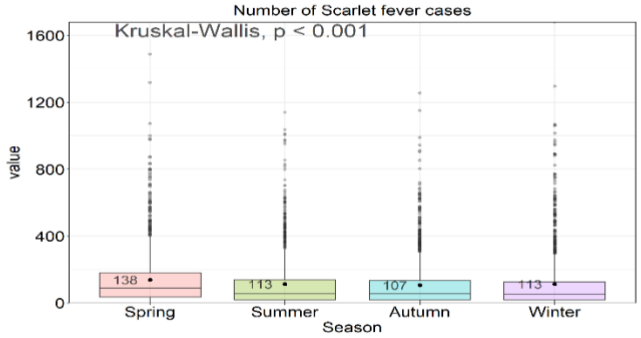

Notes: RH= Relative humidity; Ap=Air pressure; WS=Wind speed.

Supplementary Figure 8. Time series plot of the monthly mean weather condition in high-latitude areas (≥33.4 degrees north) and low-latitude areas (<33.4 degrees north) of China, 2004-2018

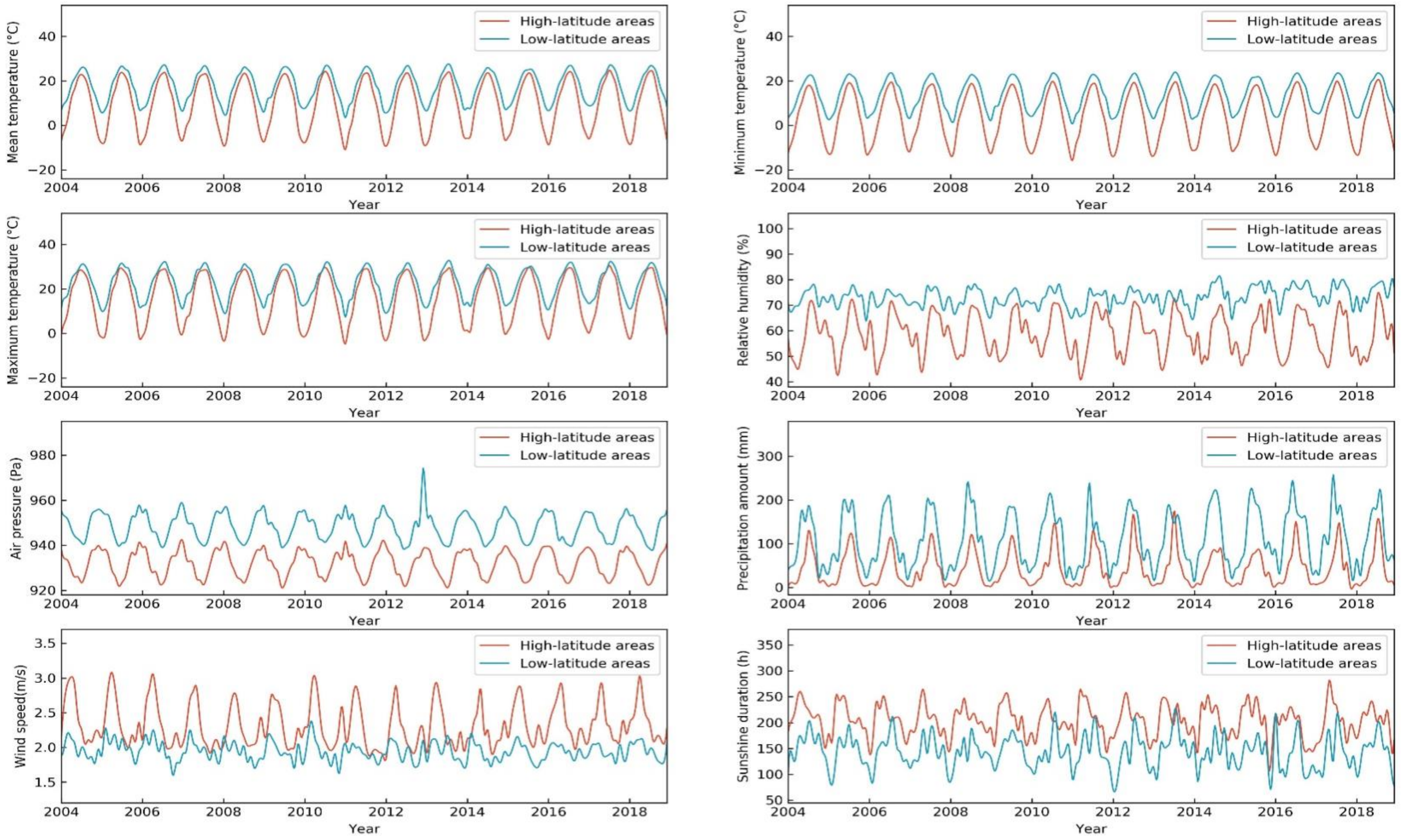

Supplementary Figure 9. Predicted exposure-response relationships in relative risk between NO_2, $PM_{2.5}$, and PM_{10} (percentiles) and scarlet fever incidence in China, 2013-2018

a. NO_2 vs. Population density

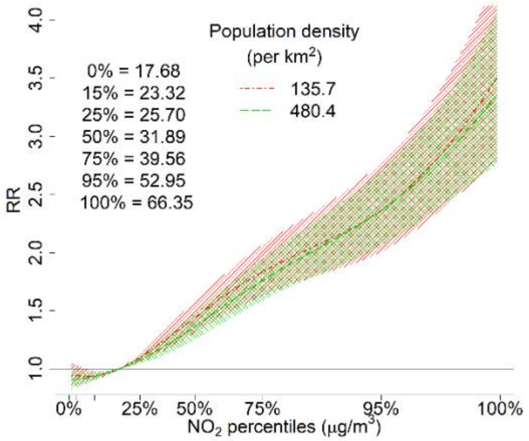

b. $PM_{2.5}$ vs. Incidence

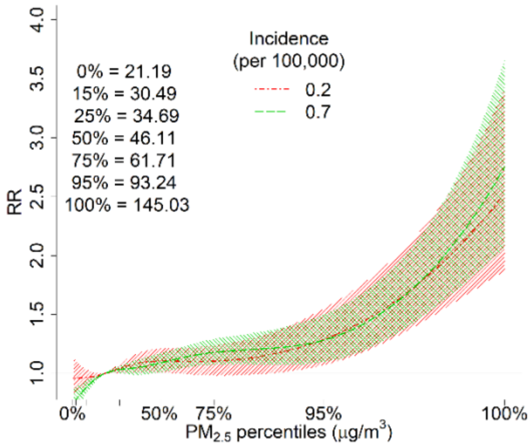

c. PM_{10} vs. Incidence

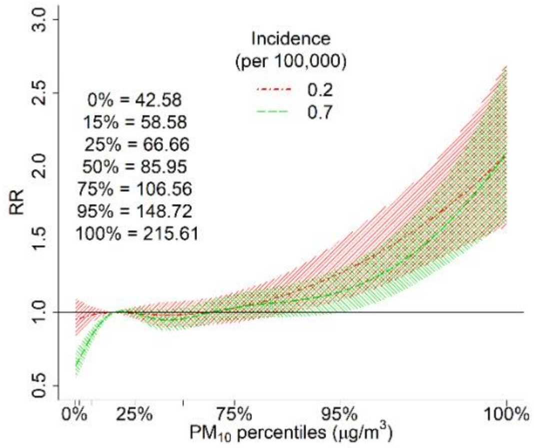

Notes: The predictive exposure-response relationships were modeled by two-stage methods including distributed lag non-linear model (DLNM) and multivariate meta-analysis. The reference concentrations were set at the 15th percentile of the air pollutants: 23.22 µg/m3 for NO_2, 30.49 µg/m3 for $PM_{2.5}$ and 58.58 µg/m3 for PM_{10}. The prediction values were set at the 25th and 75th percentile of population density and average incidence rate. Eight provinces were detected as hotspots and classified as high-incidence areas and the remaining provinces were classified as low-incidence areas. Those eight high-incidence provinces included Inner Mongolia, Jilin, Liaoning, Beijing, Tianjin, Hebei, Heilongjiang and Shandong. Reference is set at the 15th percentile of concentration.

Supplementary Figure 10. Predicted exposure-response relationships in relative risk between monthly wind speed, precipitation, sunlight (percentiles) and scarlet fever incidence before and after 2011

a. Precipitation b. Wind Speed c. Sunlight

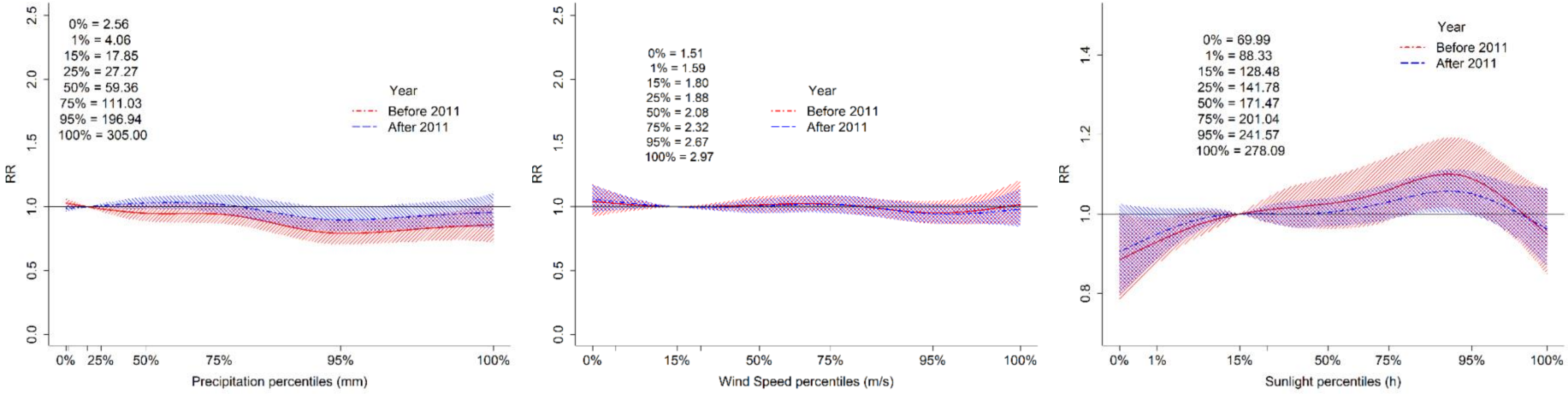

Notes:

a. Excludes three provinces including Tianjin and Beijing because of extreme outliers and Hainan because of many zero cases in months

b. Excludes Hainan because of many zero cases in months

c. Excludes Hainan because of many zero cases in months

Reference is set at the 15th percentile.

Supplementary Figure 11. Time series plot of the monthly mean NO_2 pollution concentrations and scarlet fever incidence in the overall population in China, January of 2013 to March of 2020

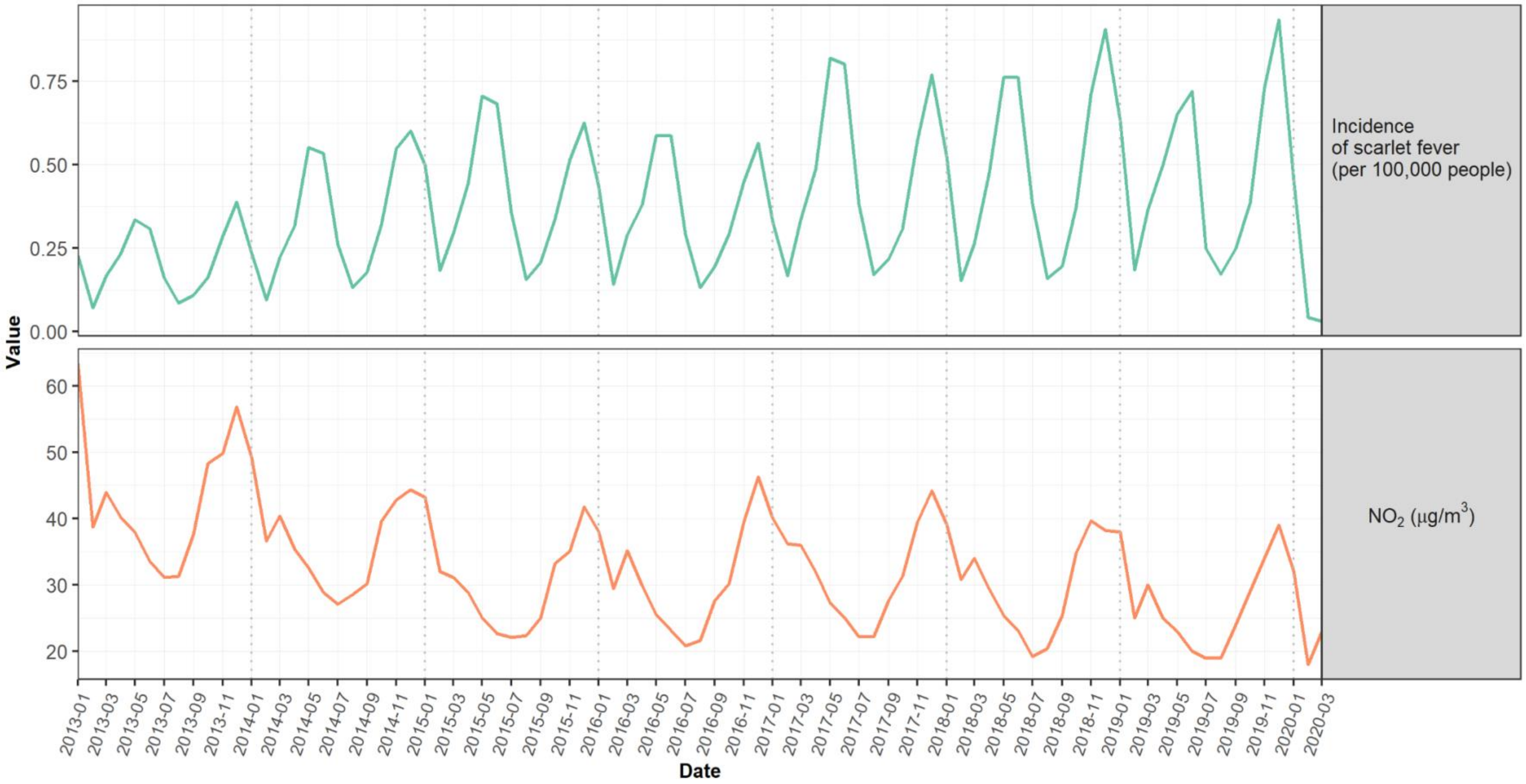

Supplementary Figure 12. The spatial distribution of average population in 2018 in 31 provinces of China

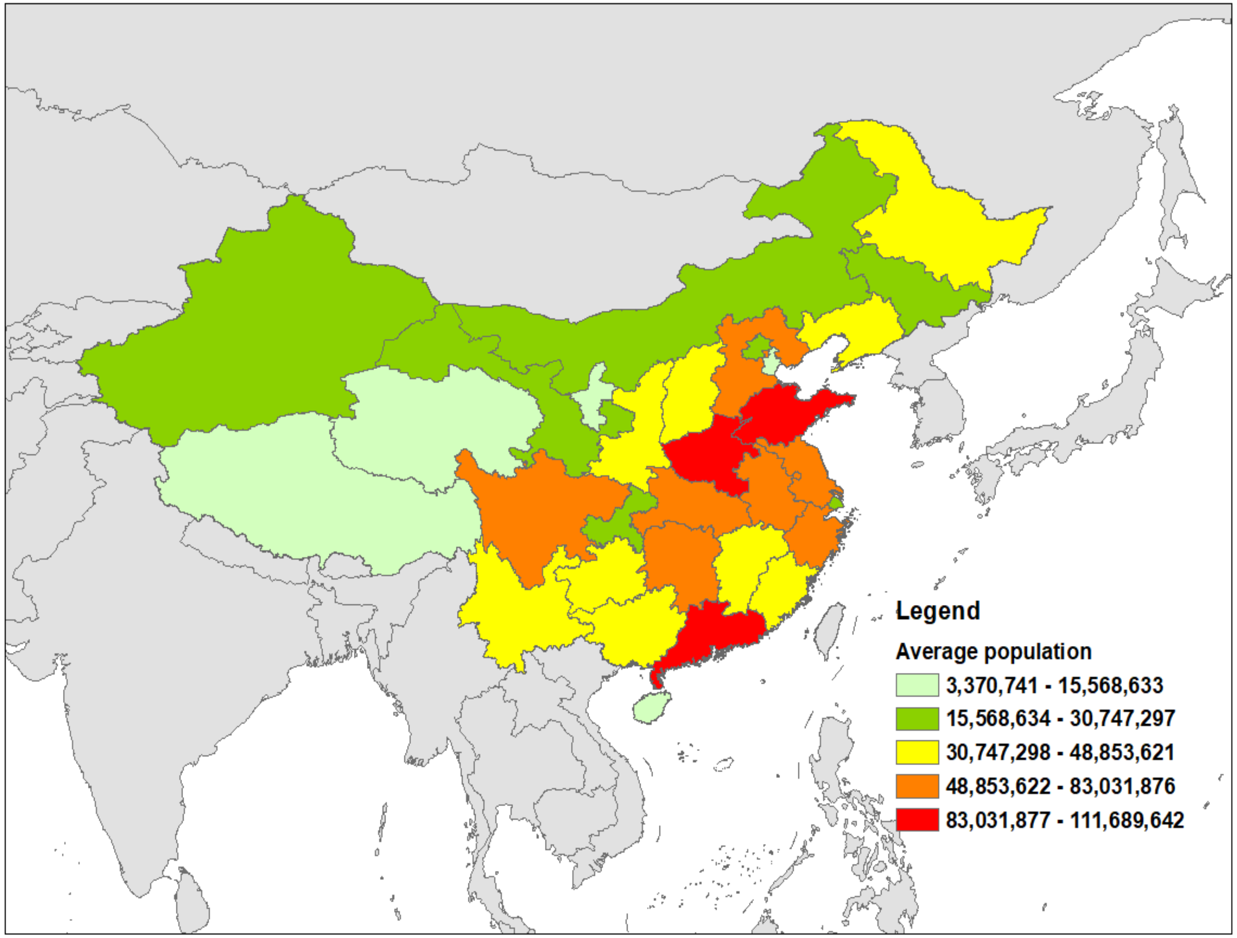

Notes: Choropleth maps of the average population, by region, in China in 2018.

Supplementary Figure 13. Geographical location of air quality monitoring stations (during 2013-2018) and meteorological monitoring stations (2004-2018) in China

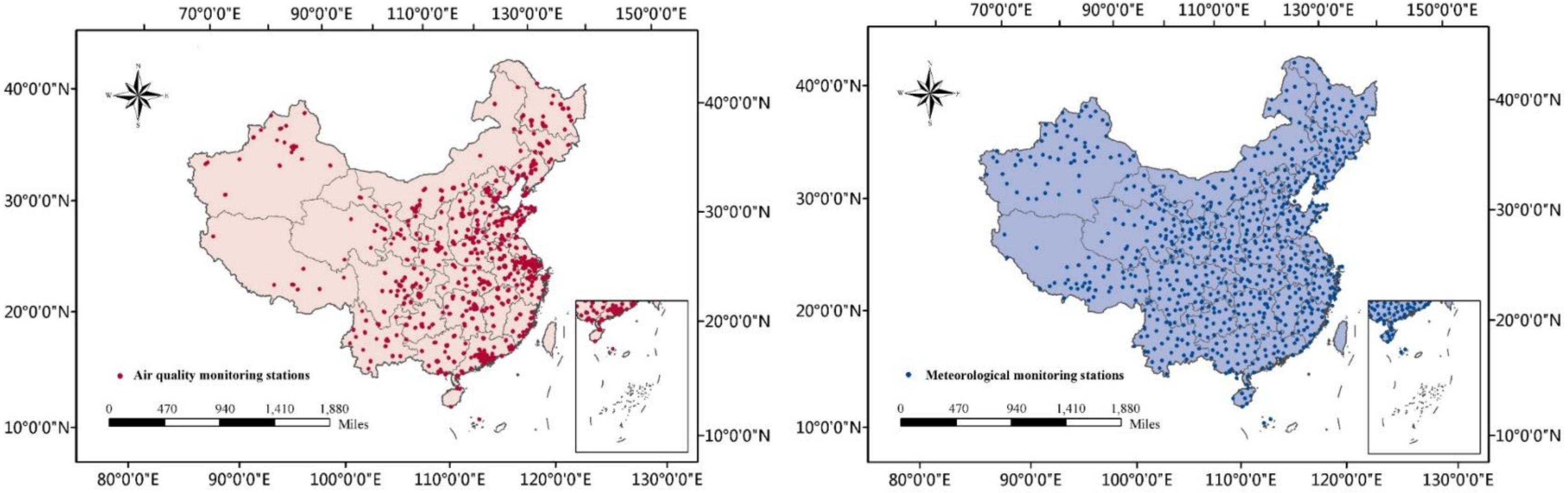

a

b

Notes: 1a: air quality monitoring stations (N=1498);

1b: meteorological monitoring stations (N=756).

Supplementary Table 1. Basic comparison of monthly air pollutants in (≥33.4 degrees north) and low-latitude areas (<33.4 degrees north) of China during 2013-2018

Pollution concentration (Monthly)	Mean (high-latitude areas)	Mean (low-latitude areas)	P value
$PM_{2.5}$ (μg/m3)	58.6213	44.3924	<0.001
PM_{10} (μg/m3)	110.8157	71.9427	<0.001
SO_2 (μg/m3)	31.8667	17.3056	<0.001
NO_2 (μg/m3)	37.0611	30.4193	<0.001
O_3 (μg/m3)	88.2222	83.9184	0.3473
CO (mg/m3)	1.2255	0.9360	<0.001

Notes: In the stratified analyses, the mean concentration of five air pollutants in high-latitude regions was much higher than in low-latitude regions (all p<0.05).

Supplementary Table 2. Basic comparison of monthly meteorological factors in high-latitude areas (≥33.4 degrees north) and low-latitude areas (<33.4 degrees north) during 2004–2010 and 2011–2018 periods

**Weather conditions (Monthly)**	**Mean (high-latitude areas)**	**Mean (low-latitude areas)**	**P value**
Mean temperature (°C)	9.2226	17.3377	<0.001
Relative humidity (%)	58.4727	72.8118	<0.001
Air pressure (Pa)	931.7705	951.2538	<0.001
Precipitation amount (mm)	42.3517	110.4676	<0.001
Wind speed (m/s)	2.3212	1.9679	<0.001
Sunlight (h)	203.3878	146.3823	<0.001

Supplementary Table 3. Cumulative relative risk and 95% confidence interval for the association between a 10 μg/m^3 increase in NO_2 and O_3 and scarlet fever incidence at lag 0 to 15 months in China, 2013-2018

Air pollutants	Single-pollutant model[a]	Multiple variables model[b]
NO_2	1.01 (0.97-1.04)	1.06 (1.02-1.10)
O_3	1.04 (1.01-1.07)	1.04 (1.01-1.07)

Notes: The reference values are set as 40 μg/$m^3$and 160 μg/m^3 for NO_2 and O_3 based on China guideline II.

[a] The single poulltant models consider not only the interested air pollutants but also consider the temporal trend, quantile groups for average incidences and incidence in the previous month.

[b] The mutple variables models consider not only the interested air pollutants but also consider other air pollutants (NO_2 and O_3), meteorological conditions (sunlight, wind speed, relative humidity, precipitation and mean temperature), the other adjusted variables in the model are temporal trend, the indicator variable of summer and winter breaks, quantile groups for average incidences, and incidence in the previous month.

Supplementary Table 4. Scarlet fever case definition and classification in China

**Scarlet fever classification**	**Scarlet fever case definition**
A probable case of scarlet fever	A probable case of scarlet fever is defined as a patient with clinical manifestations, including fever, rash, sore throat, red tonsils, and enlarged lymph nodes, consistent with the total number of leukocytes and neutrophils increasing or with possible toxic granulation in routine blood tests.
A clinically diagnosed case	A clinically diagnosed case is defined as a probable case, additionally, with any one of the following (1) *Group A Streptococcus* by rapid antigen detection is positive; (2) The result of bacteria identification is *β hemolytic streptococcus* by bacterial culture and by microscopy. (3) The result of a bacitracin-sensitive test is positive; (4) The result of biochemical identification is *Streptococcus pyogenes.*
A confirmed case	A confirmed case is defined as a probable case with, additionally, laboratory evidence of *group A streptococcal (GAS) infection.*

Notes: Scarlet fever case definition and classification in China based on national diagnostic criteria (WS282-2008 and GB15993-1995) promulgated by the Health Ministry of China.
