# Supplementary material for: Exposure to air pollution and scarlet fever resurgence in China: a six-year surveillance study
Source: Nat Commun. 2020 Aug 25;11:4229. doi: 10.1038/s41467-020-17987-8 (PMC7447791; doi:10.1038/s41467-020-17987-8)
Supplement: Supplementary file 3 — Reporting Summary [file 41467_2020_17987_MOESM3_ESM.pdf]

nature research

Corresponding author(s): Shelan Liu

Last updated by author(s): Jul 20, 2020

Reporting Summary

Nature Research wishes to improve the reproducibility of the work that we publish. This form provides structure for consistency and transparency in reporting. For further information on Nature Research policies, see our Editorial Policies and the Editorial Policy Checklist.

Statistics

For all statistical analyses, confirm that the following items are present in the figure legend, table legend, main text, or Methods section.

n/a	Confirmed	
☐	☒	The exact sample size (n) for each experimental group/condition, given as a discrete number and unit of measurement
☐	☒	A statement on whether measurements were taken from distinct samples or whether the same sample was measured repeatedly
☐	☒	The statistical test(s) used AND whether they are one- or two-sided<br>*Only common tests should be described solely by name; describe more complex techniques in the Methods section.*
☐	☒	A description of all covariates tested
☐	☒	A description of any assumptions or corrections, such as tests of normality and adjustment for multiple comparisons
☐	☒	A full description of the statistical parameters including central tendency (e.g. means) or other basic estimates (e.g. regression coefficient) AND variation (e.g. standard deviation) or associated estimates of uncertainty (e.g. confidence intervals)
☐	☒	For null hypothesis testing, the test statistic (e.g. F, t, r) with confidence intervals, effect sizes, degrees of freedom and P value noted<br>*Give P values as exact values whenever suitable.*
☒	☐	For Bayesian analysis, information on the choice of priors and Markov chain Monte Carlo settings
☒	☐	For hierarchical and complex designs, identification of the appropriate level for tests and full reporting of outcomes
☐	☒	Estimates of effect sizes (e.g. Cohen's d, Pearson's r), indicating how they were calculated

Our web collection on statistics for biologists contains articles on many of the points above.

Software and code

Policy information about availability of computer code

Data collection	We collected the data into two major formats including Microsoft Excel and csv format. Before, we conducted spatial analysis. We used ESRI ArcGIS software to convert the attribute data into shapefile format.
Data analysis	We primarily used R software for data analysis and used ESRI ArcGIS for spatial analysis, making ring map and thematic maps.

For manuscripts utilizing custom algorithms or software that are central to the research but not yet described in published literature, software must be made available to editors and reviewers. We strongly encourage code deposition in a community repository (e.g. GitHub). See the Nature Research guidelines for submitting code & software for further information.

Data

Policy information about availability of data

All manuscripts must include a data availability statement. This statement should provide the following information, where applicable:

- Accession codes, unique identifiers, or web links for publicly available datasets
- A list of figures that have associated raw data
- A description of any restrictions on data availability

The dataset and R codes used in this study have been deposited in an open archive, figshare.com (10.6084/m9.figshare.12237596).

Field-specific reporting

Ecological, evolutionary & environmental sciences study design

All studies must disclose on these points even when the disclosure is negative.

Study description	This study aimed to investigate the association and mechanism between the resurgence of scarlet fever and long-term exposure to air pollutants and meteorological conditions in China nationwide. In a retrospective multicenter study, we assessed 655,039 scarlet fever cases across 31 province-level administrative divisions of China.
Research sample	Four sources of data were combined to be collected throughout China. The first was obtained from the official website of the National Health Commission of the People's Republic of China (http://www.nhc.gov.cn/jkj/s3578/202004/b1519e1bc1a944fc8ec176db600f68d1.shtml) and health commissions at the province level. The second was the Chinese open access notifiable infectious disease report database (available from the Chinese Public Health Science Data Center). The third was from the Notifiable Infectious Disease Surveillance System (NNIDSS) covering the period from January 1, 2004 to December 31, 2018. Fourth, population data were from the National Bureau of Statistics of the People's Republic of China and are updated at the end of every year.
Sampling strategy	This is a nationwide disease surveillance data in China from 2004 to 2018.
Data collection	Data collection is from four different data sources.
Timing and spatial scale	The temporal resolution is the month and the spatial resolution is the province. The data of scarlet fever and the weather conditions ranged from 2004 to 2018. The data of air pollution ranged from 2013 to 2018.
Data exclusions	In Supplement 10, when we modelled the exposure-response relationships in relative risk between monthly wind speed,precipitation, sunlight and scarlet fever incidence before and after 2011. There are some provinces having outliers or having many zero cases in months. Therefore, we excluded some provinces in that subgroup analysis.
Reproducibility	The raw data and the modelling R codes are listed in an open archive. The readers can reproduce them by analyzing the data we provided.
Randomization	This is a nationwide surveillance data without any sampling procedure. The data is all analyzed in the aggregated form. When we used DLNM for estimating the risks, we conducted both single-variable and multiple-variables models. In those two kinds of models, we also adjust the potential confounders in the models.
Blinding	Our data is all in aggregated format which is already blinded itself.

Did the study involve field work? ☐ Yes ☒ No

Reporting for specific materials, systems and methods

We require information from authors about some types of materials, experimental systems and methods used in many studies. Here, indicate whether each material, system or method listed is relevant to your study. If you are not sure if a list item applies to your research, read the appropriate section before selecting a response.

Materials & experimental systems

n/a	Involved in the study	
☒	☐	Antibodies
☒	☐	Eukaryotic cell lines
☒	☐	Palaeontology and archaeology
☒	☐	Animals and other organisms
☐	☒	Human research participants
☒	☐	Clinical data
☒	☐	Dual use research of concern

Methods

n/a	Involved in the study	
☒	☐	ChIP-seq
☒	☐	Flow cytometry
☒	☐	MRI-based neuroimaging

Human research participants

Policy information about studies involving human research participants

Population characteristics	There were a total of 655,039 scarlet fever cases across 31 provinces during 2004-2018. We extracted data on scarlet fever, including the number of cases, incidence, and patient data stratified by onset date (month and year) and province. The data we used is the counts and incidences without any individual demographic information.
Recruitment	This is the nationwide disease surveillance data. All the scarlet fever cases were mandated to be reported by the law. Scarlet fever is defined as a class B notifiable infectious disease based on criteria issued by the Ministry of Health of the People's Republic of China (http://ww w.chinacdc.cn /jkzt/crb/zl/xhr/zstd/200509/t20050907_24852.html). All probable, clinically diagnosed, and confirmed scarlet fever cases must be reported within 24 hours of diagnosis online to this system.
Ethics oversight	The data used in this study is all from open data. Thus, we waived from the approval of ethics committee.

Note that full information on the approval of the study protocol must also be provided in the manuscript.
