# Supplementary material for: Exposure to air pollution and scarlet fever resurgence in China: a six-year surveillance study
Source: Nat Commun. 2020 Aug 25;11:4229. doi: 10.1038/s41467-020-17987-8 (PMC7447791; doi:10.1038/s41467-020-17987-8)
Supplement: Supplementary file 4 — Source Data [file 41467_2020_17987_MOESM4_ESM.zip › SUPPLEMENT 1/Supplement 1.docx]

**Supplement 1: Scarlet fever case definition and classification**

According to WS282-2008 and GB15993-1995 promulgated by the Health Ministry of China, the case definition and classification of scarlet fever are as follows:

A probable case of scarlet fever is defined as a patient with clinical manifestations, including fever, rash, sore throat, red tonsils, and enlarged lymph nodes, consistent with the total number of leukocytes and neutrophils increasing or with possible toxic granulation in routine blood tests.

A clinically diagnosed case is defined as a probable case, additionally, with any one of the following (1) *Group A Streptococcus* by rapid antigen detection is positive; (2) The result of bacteria identification is *β hemolytic streptococcus* by bacterial culture and by microscopy. (3) The result of a bacitracin-sensitive test is positive; (4) The result of biochemical identification is *Streptococcus pyogenes.*

A confirmed case is defined as a probable case with, additionally, laboratory evidence of *group A streptococcal (GAS) infection.*
