# Supplementary figures and images for: Exposure to air pollution and scarlet fever resurgence in China: a six-year surveillance study
Source: Nat Commun. 2020 Aug 25;11:4229. doi: 10.1038/s41467-020-17987-8 (PMC7447791; doi:10.1038/s41467-020-17987-8)

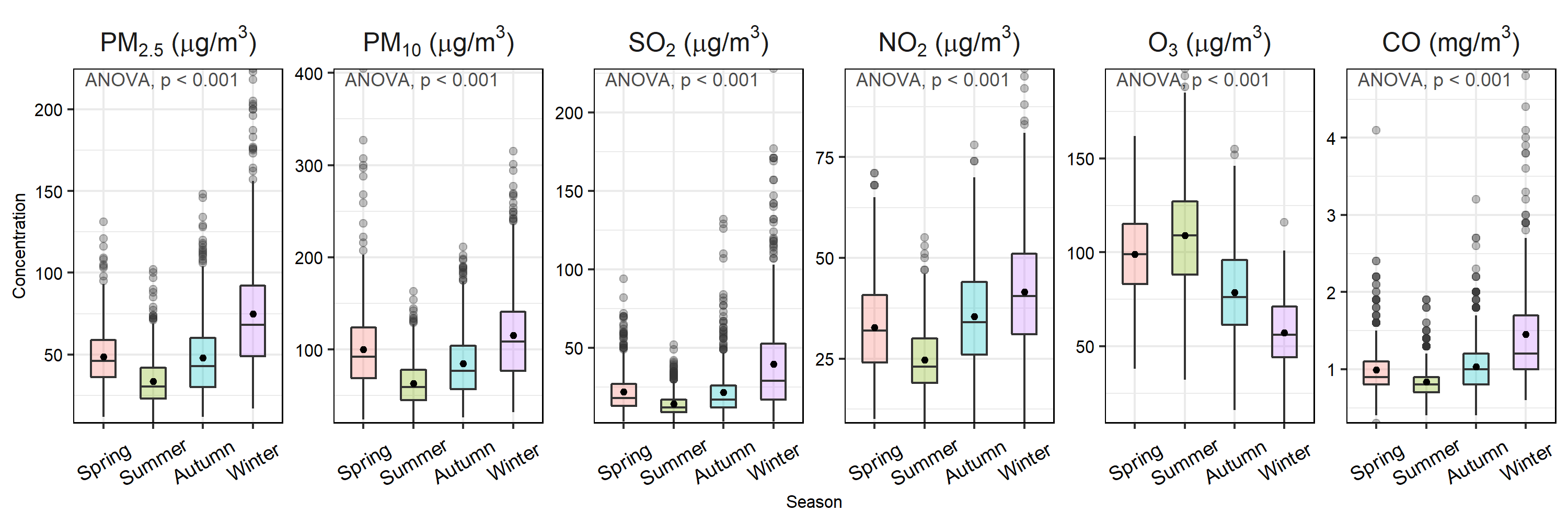

Supplement: Supplementary file 4 — Source Data [file 41467_2020_17987_MOESM4_ESM.zip › FIGURE 2/FIGURE_2A.tiff]

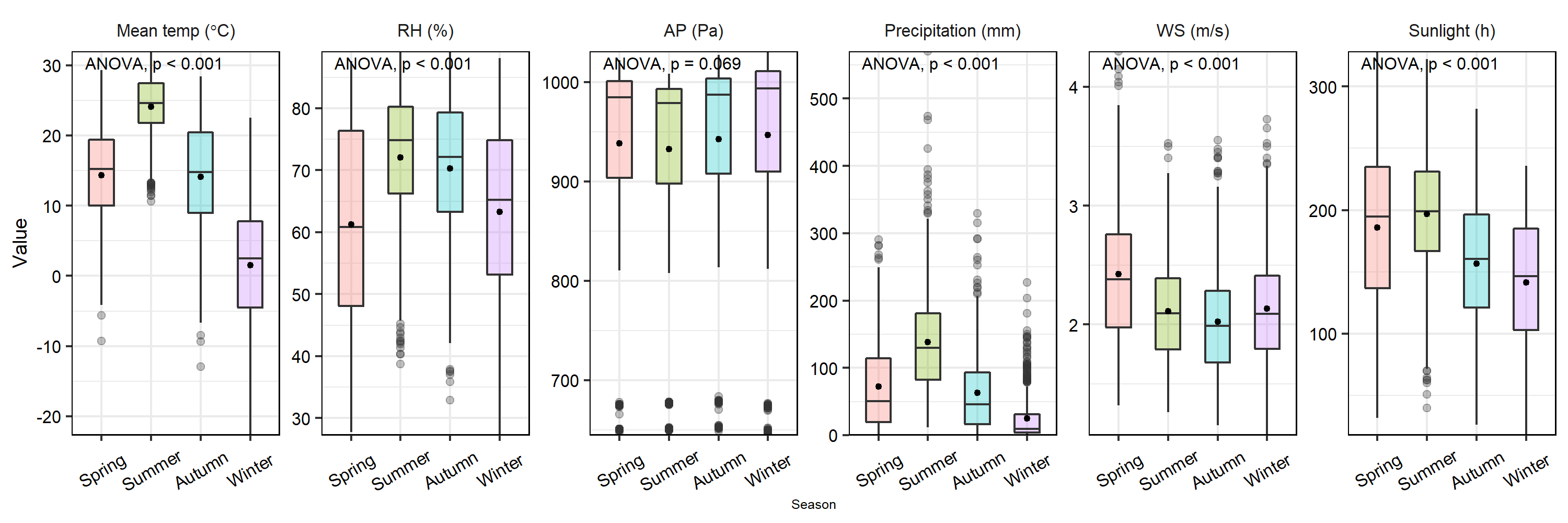

Supplement: Supplementary file 4 — Source Data [file 41467_2020_17987_MOESM4_ESM.zip › FIGURE 2/FIGURE_2B.tiff]

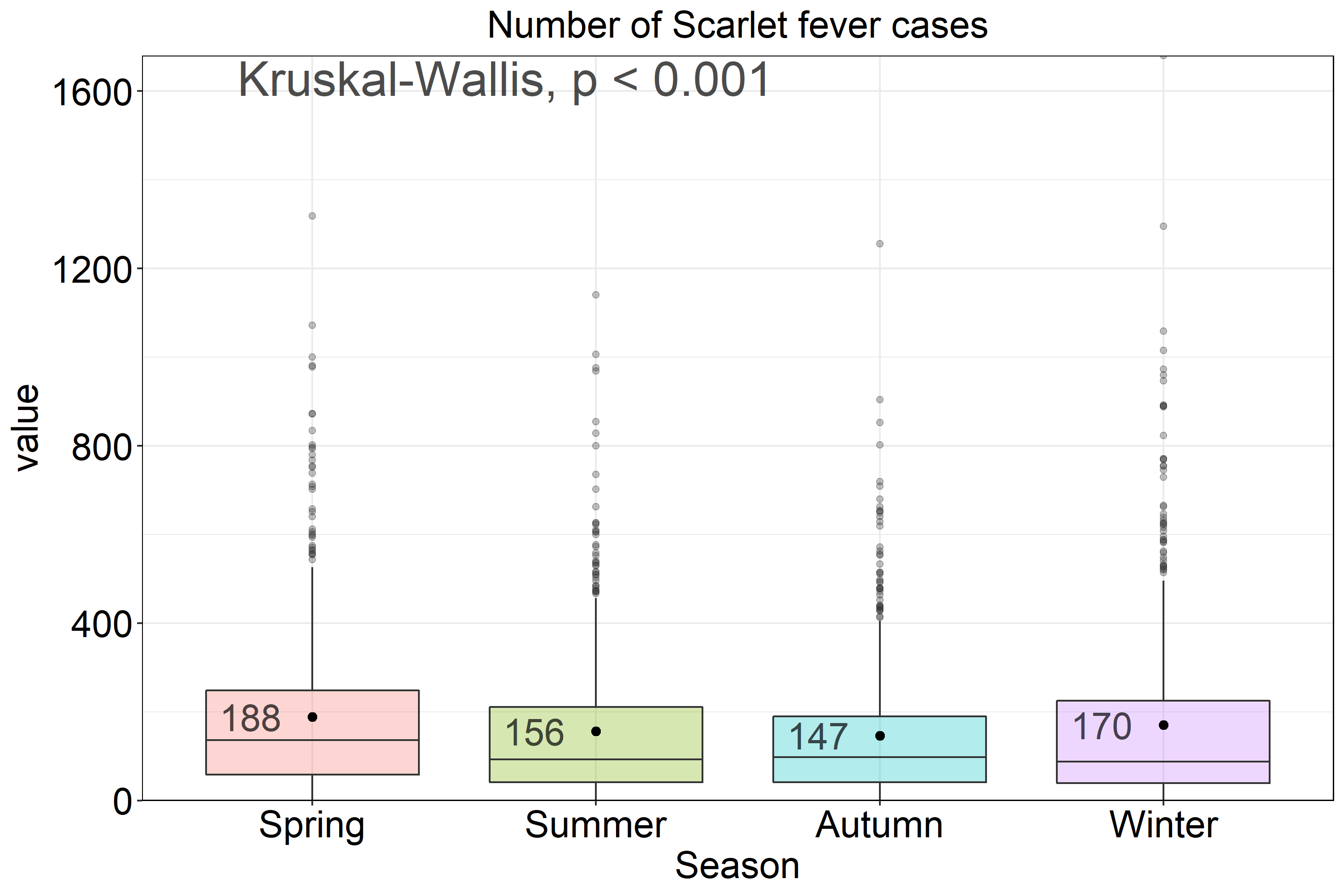

Supplement: Supplementary file 4 — Source Data [file 41467_2020_17987_MOESM4_ESM.zip › FIGURE 2/FIGURE_2C.tiff]

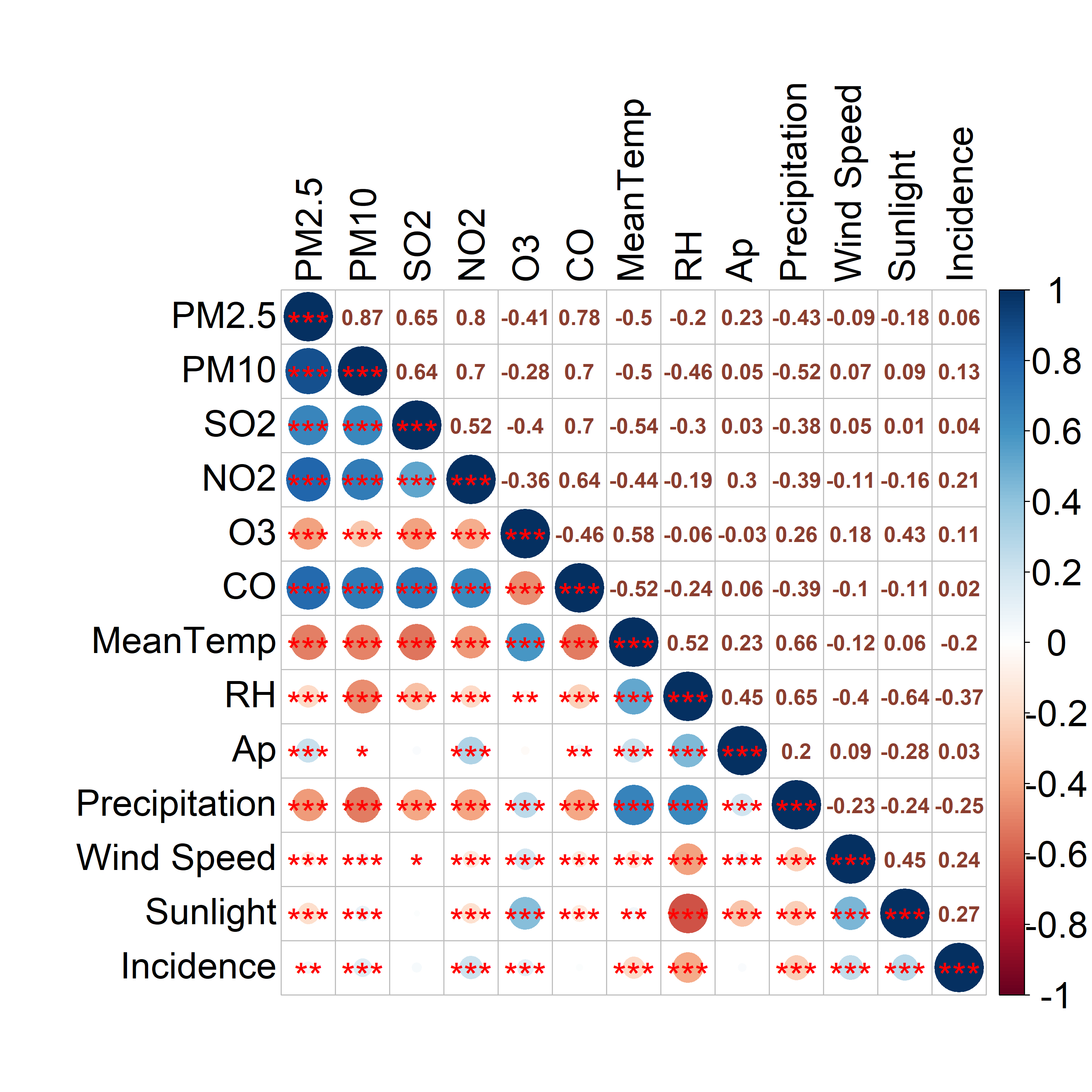

Supplement: Supplementary file 4 — Source Data [file 41467_2020_17987_MOESM4_ESM.zip › FIGURE 4/correlationplot.tiff]

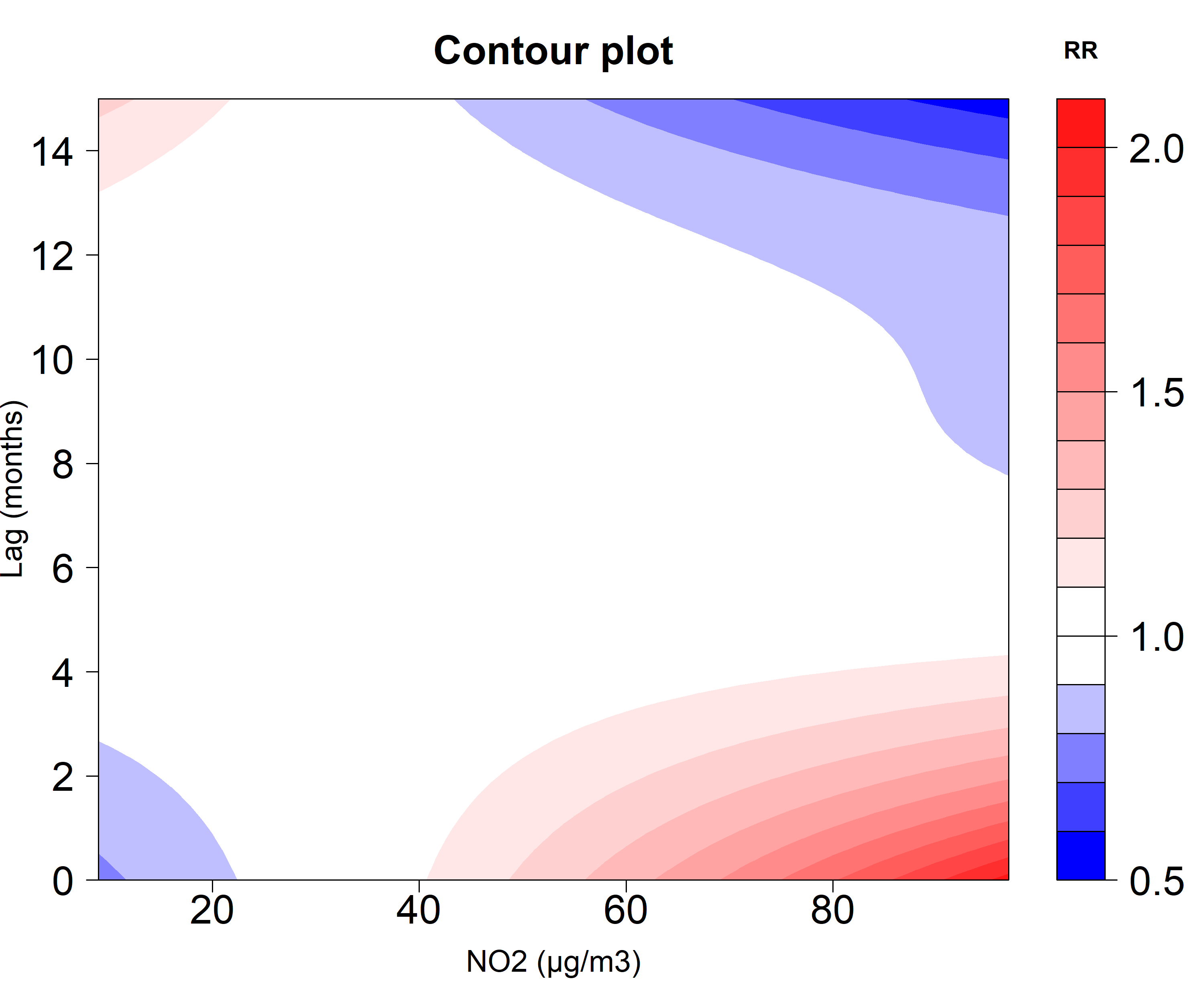

Supplement: Supplementary file 4 — Source Data [file 41467_2020_17987_MOESM4_ESM.zip › FIGURE 5/(A)-(D)/FIGURE_5A.tiff]

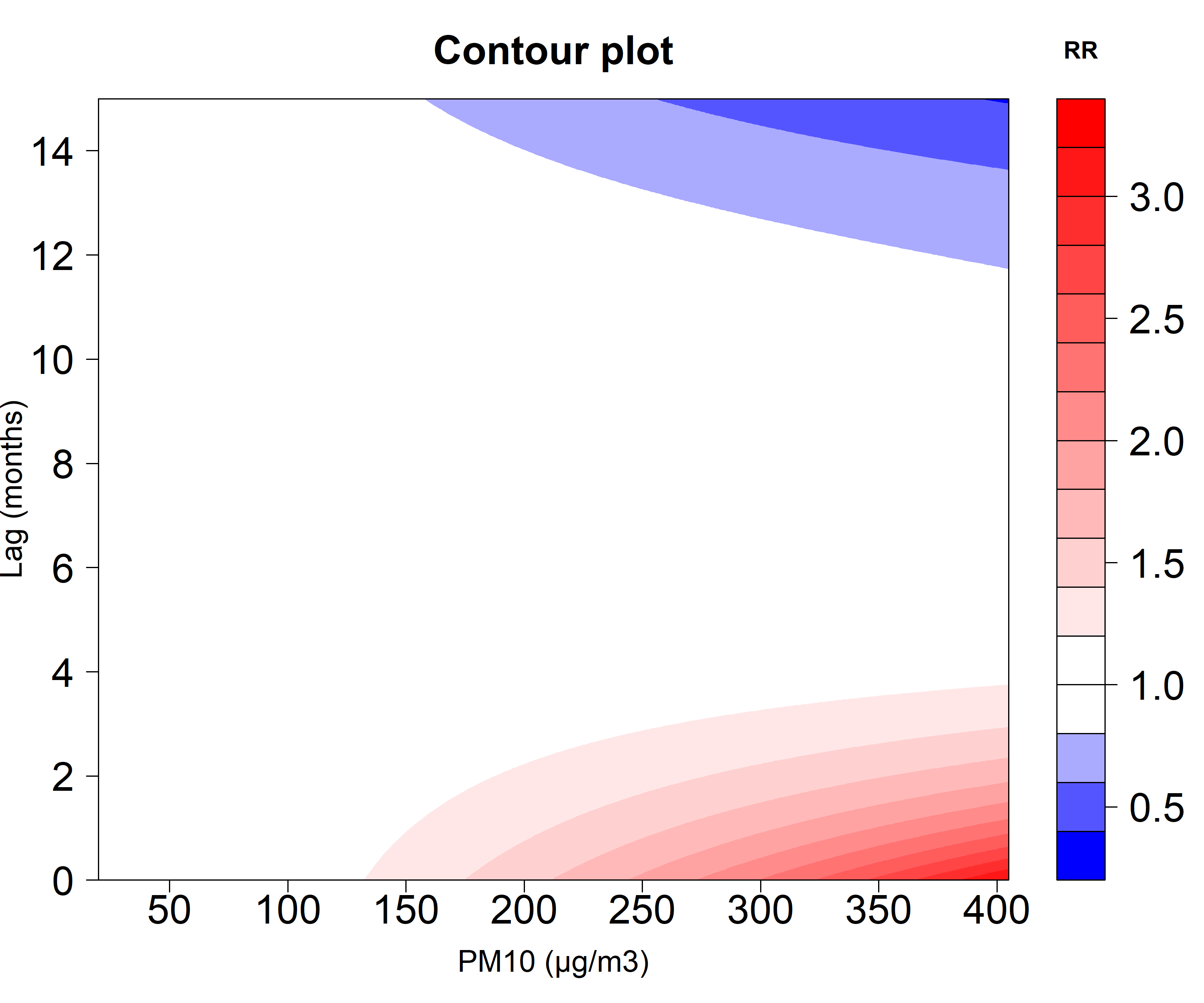

Supplement: Supplementary file 4 — Source Data [file 41467_2020_17987_MOESM4_ESM.zip › FIGURE 5/(A)-(D)/FIGURE_5B.tiff]

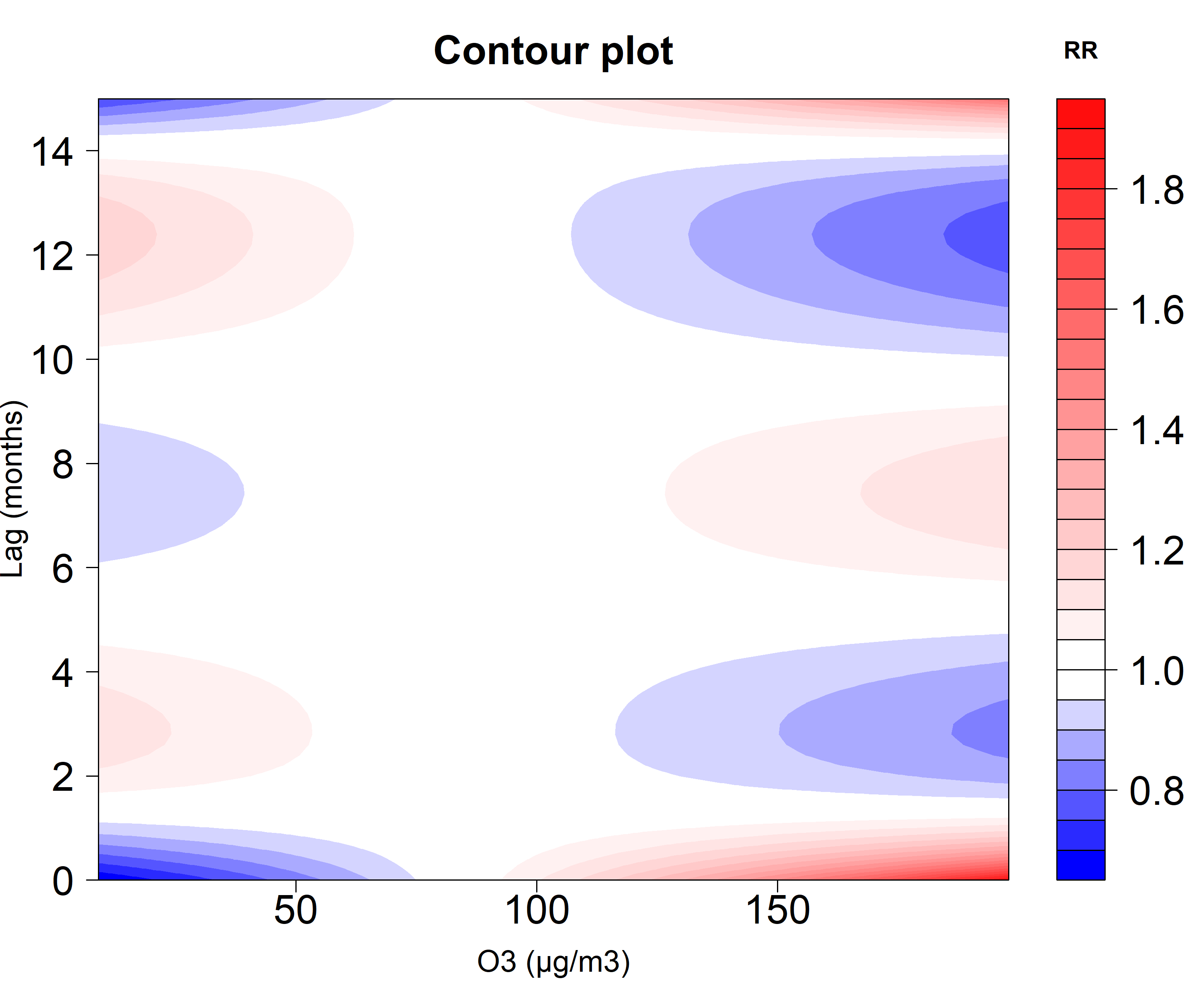

Supplement: Supplementary file 4 — Source Data [file 41467_2020_17987_MOESM4_ESM.zip › FIGURE 5/(A)-(D)/FIGURE_5C.tiff]

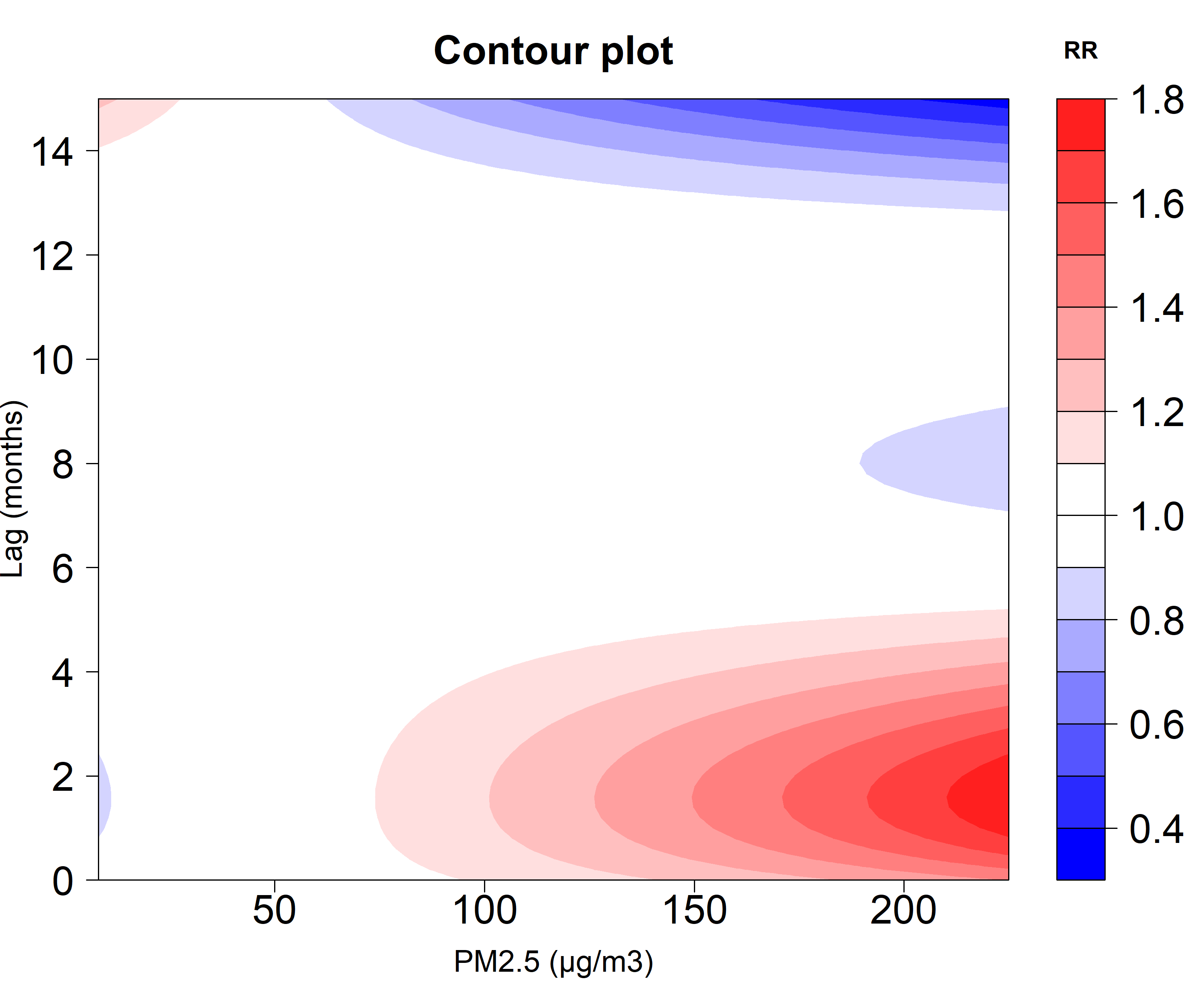

Supplement: Supplementary file 4 — Source Data [file 41467_2020_17987_MOESM4_ESM.zip › FIGURE 5/(A)-(D)/FIGURE_5D.tiff]

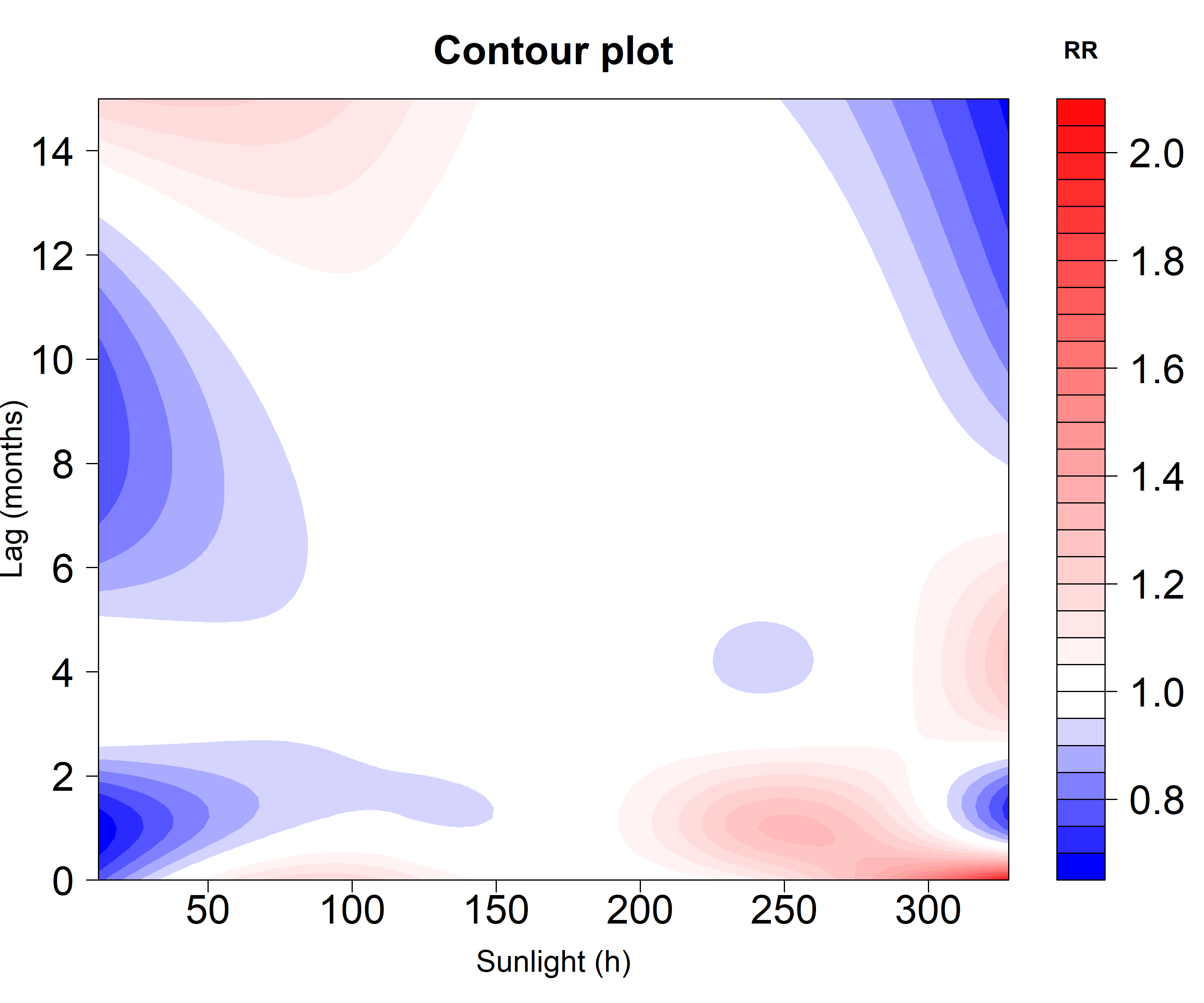

Supplement: Supplementary file 4 — Source Data [file 41467_2020_17987_MOESM4_ESM.zip › FIGURE 5/(E)-(I)/FIGURE_5E.tiff]

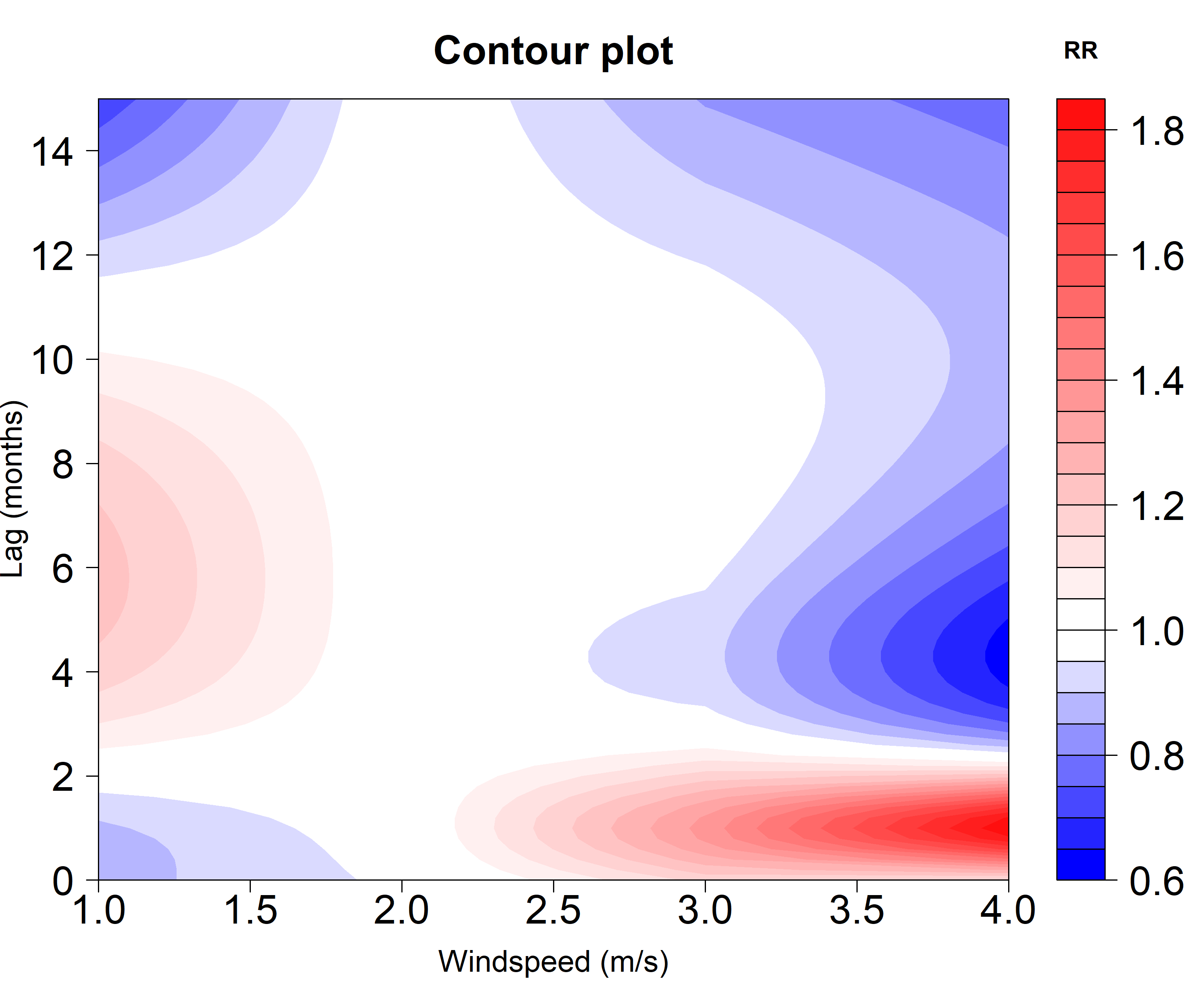

Supplement: Supplementary file 4 — Source Data [file 41467_2020_17987_MOESM4_ESM.zip › FIGURE 5/(E)-(I)/FIGURE_5F.tiff]

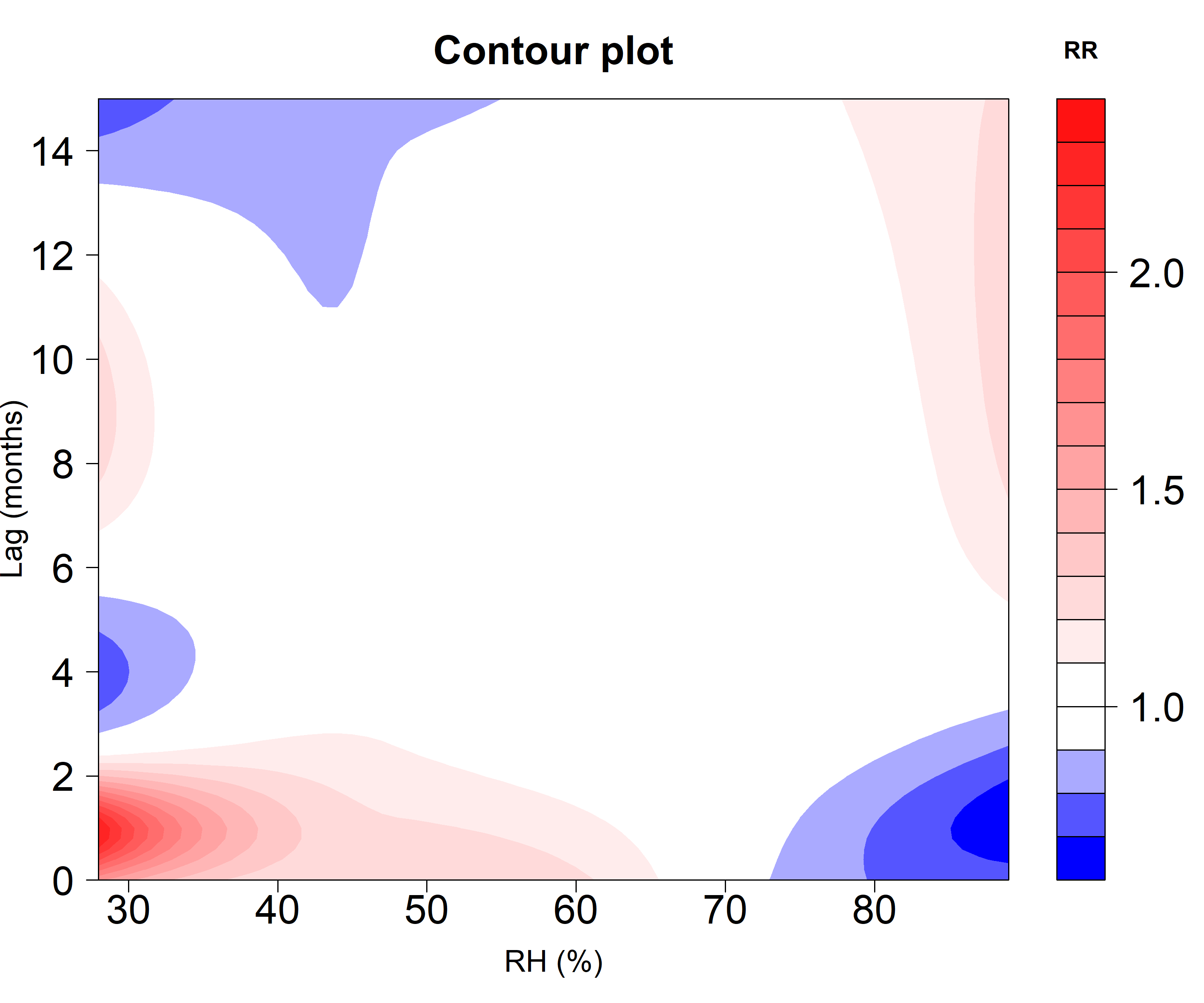

Supplement: Supplementary file 4 — Source Data [file 41467_2020_17987_MOESM4_ESM.zip › FIGURE 5/(E)-(I)/FIGURE_5G.tiff]

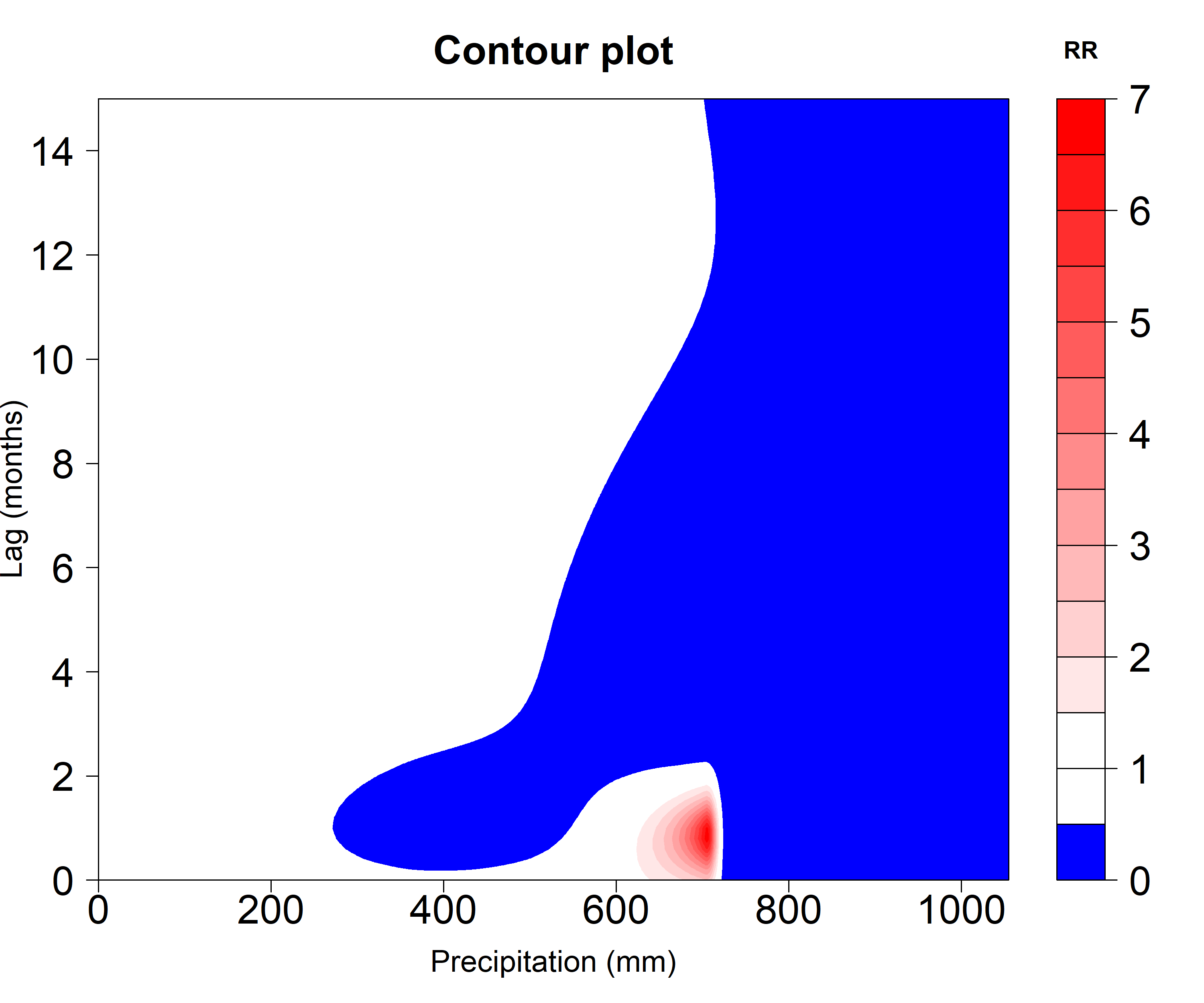

Supplement: Supplementary file 4 — Source Data [file 41467_2020_17987_MOESM4_ESM.zip › FIGURE 5/(E)-(I)/FIGURE_5H.tiff]

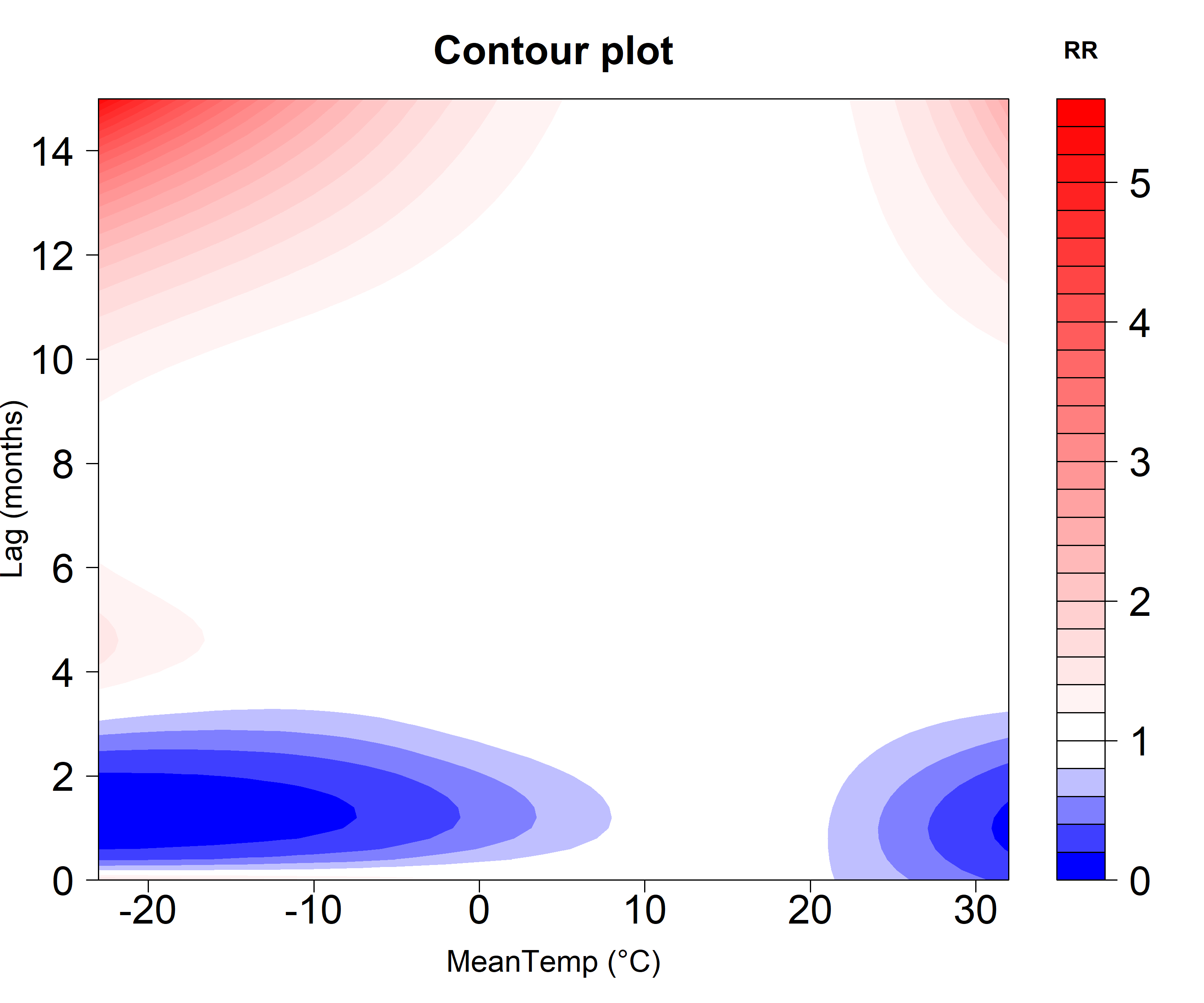

Supplement: Supplementary file 4 — Source Data [file 41467_2020_17987_MOESM4_ESM.zip › FIGURE 5/(E)-(I)/FIGURE_5I.tiff]

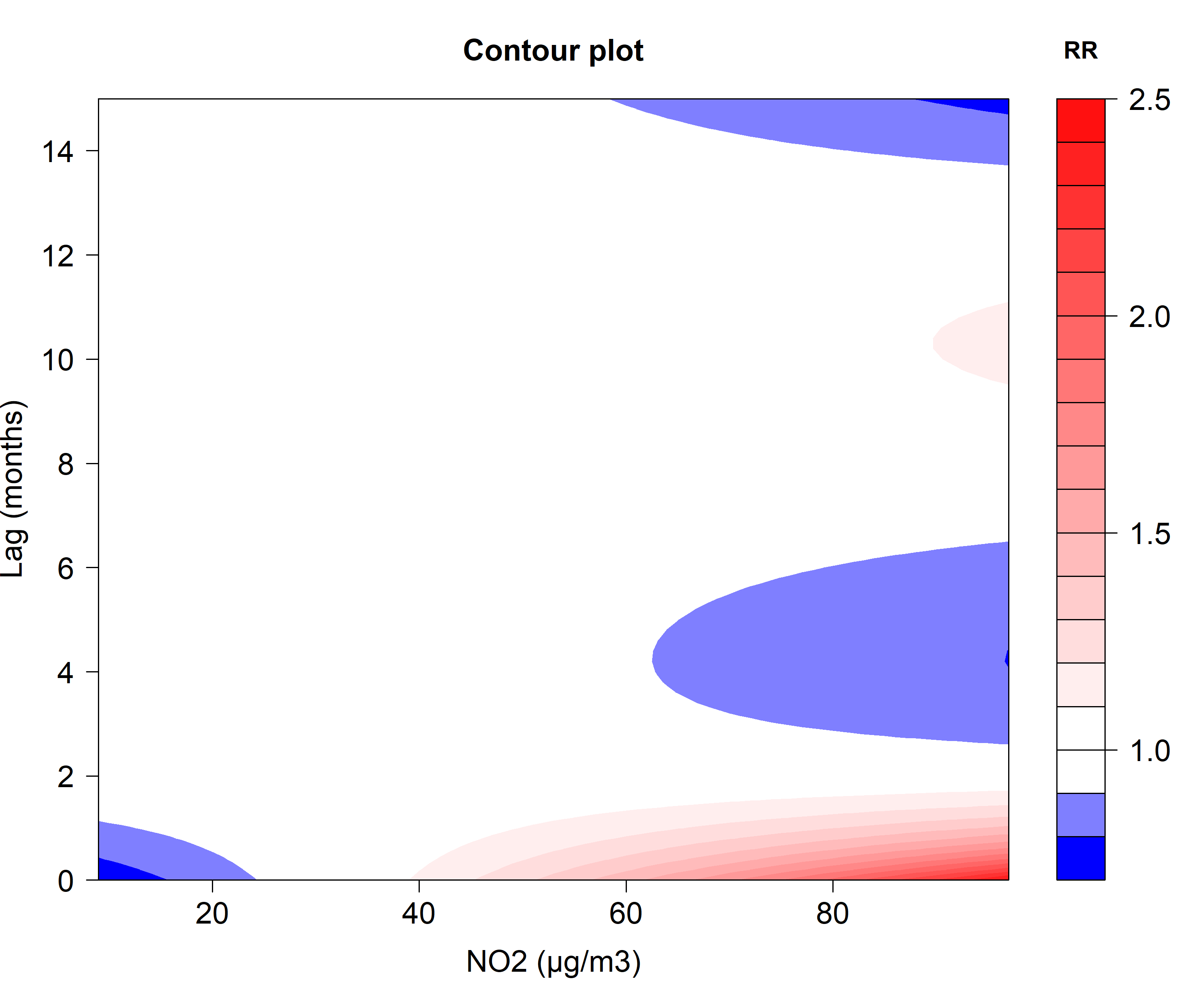

Supplement: Supplementary file 4 — Source Data [file 41467_2020_17987_MOESM4_ESM.zip › FIGURE 6/FIGURE_6A.tiff]

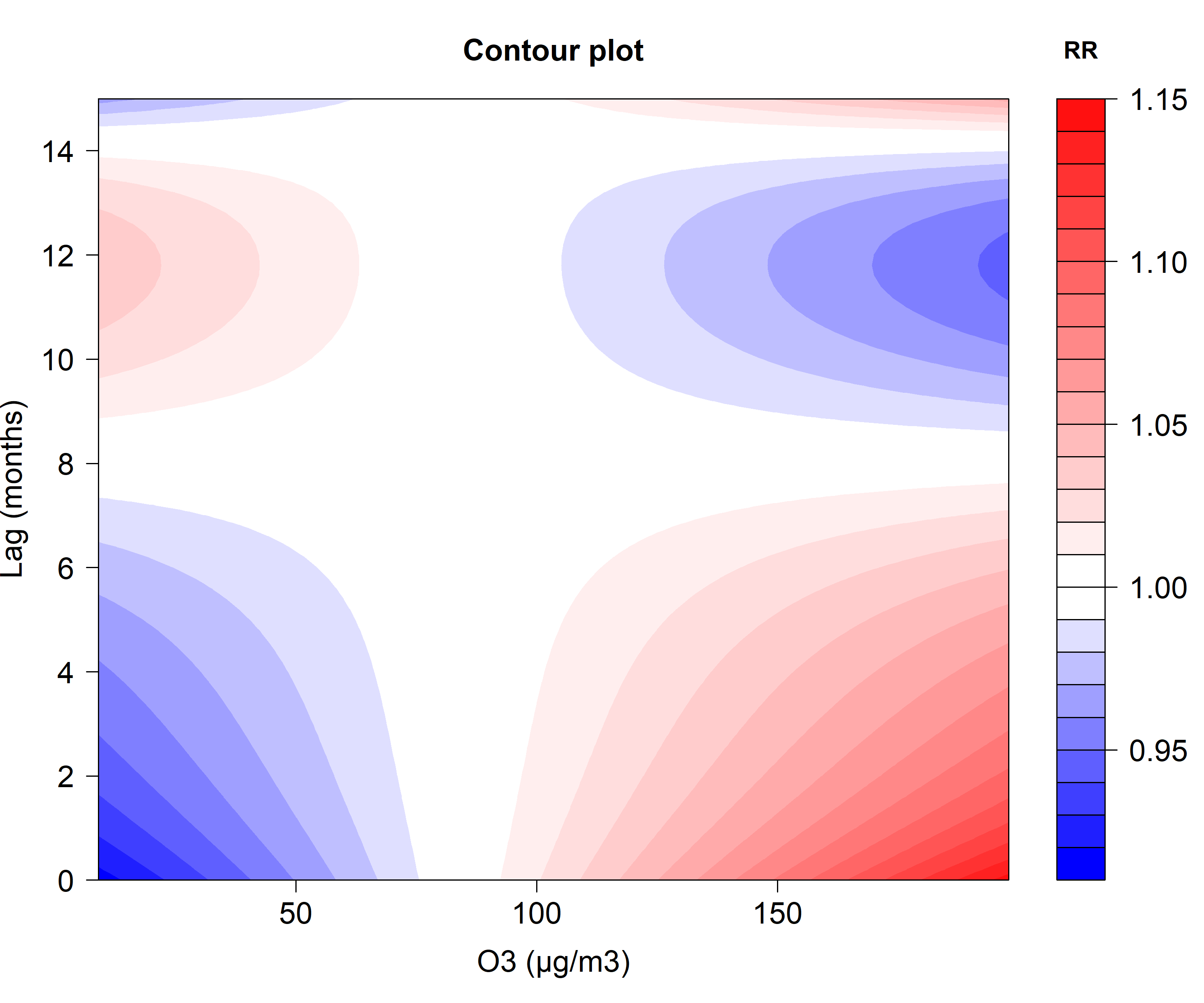

Supplement: Supplementary file 4 — Source Data [file 41467_2020_17987_MOESM4_ESM.zip › FIGURE 6/FIGURE_6B.tiff]

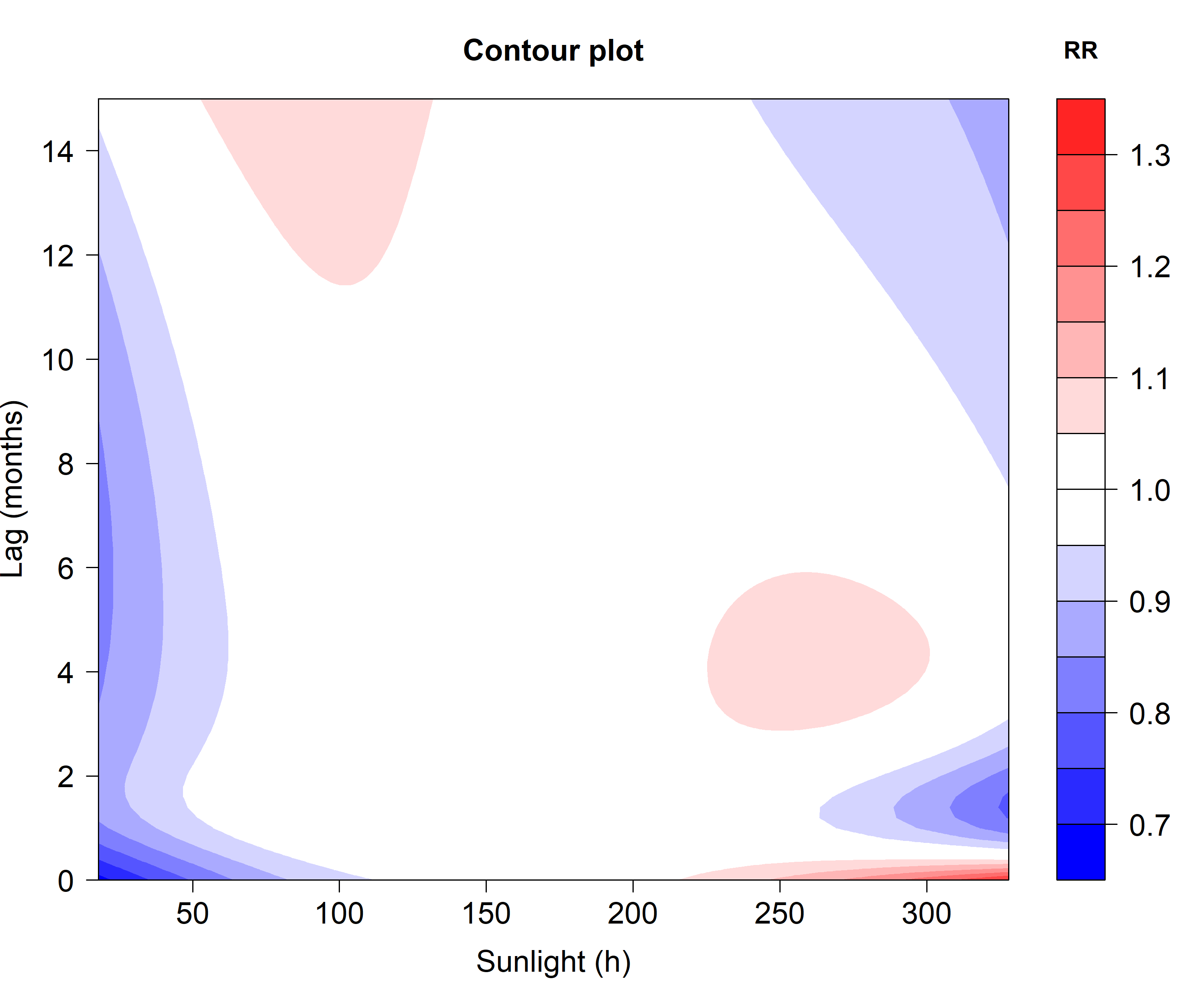

Supplement: Supplementary file 4 — Source Data [file 41467_2020_17987_MOESM4_ESM.zip › FIGURE 6/FIGURE_6C.tiff]

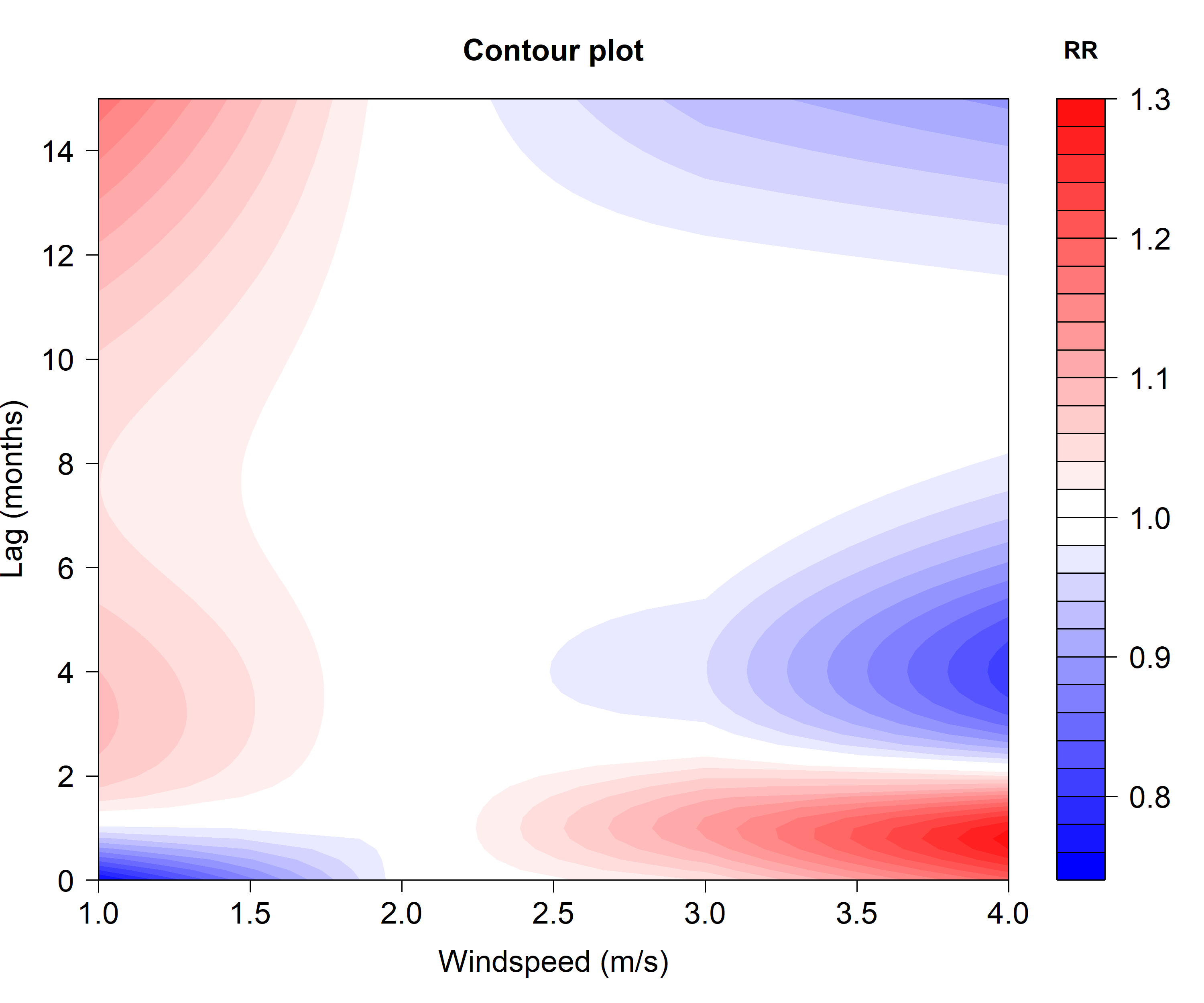

Supplement: Supplementary file 4 — Source Data [file 41467_2020_17987_MOESM4_ESM.zip › FIGURE 6/FIGURE_6D.tiff]

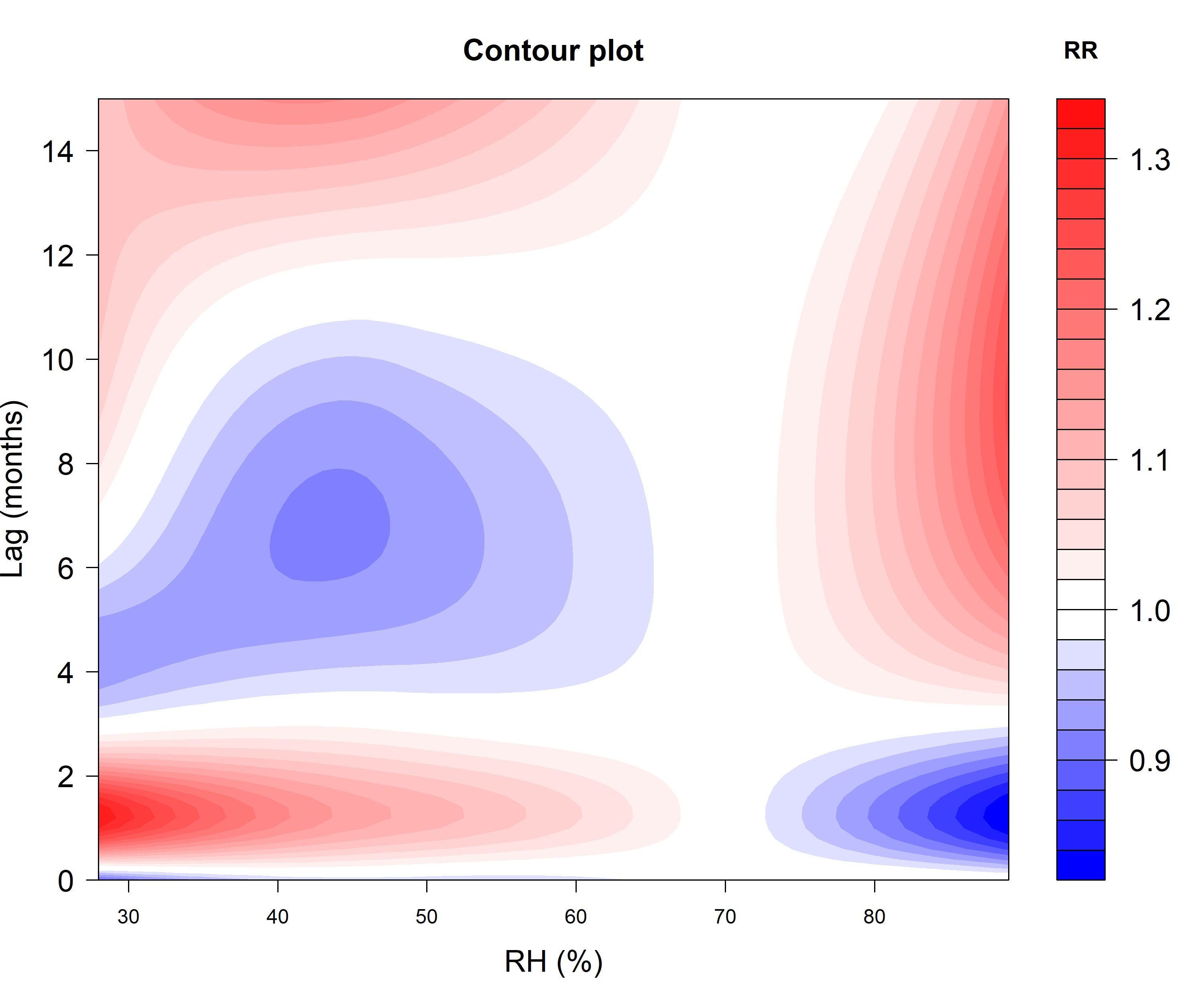

Supplement: Supplementary file 4 — Source Data [file 41467_2020_17987_MOESM4_ESM.zip › FIGURE 6/FIGURE_6E.tiff]

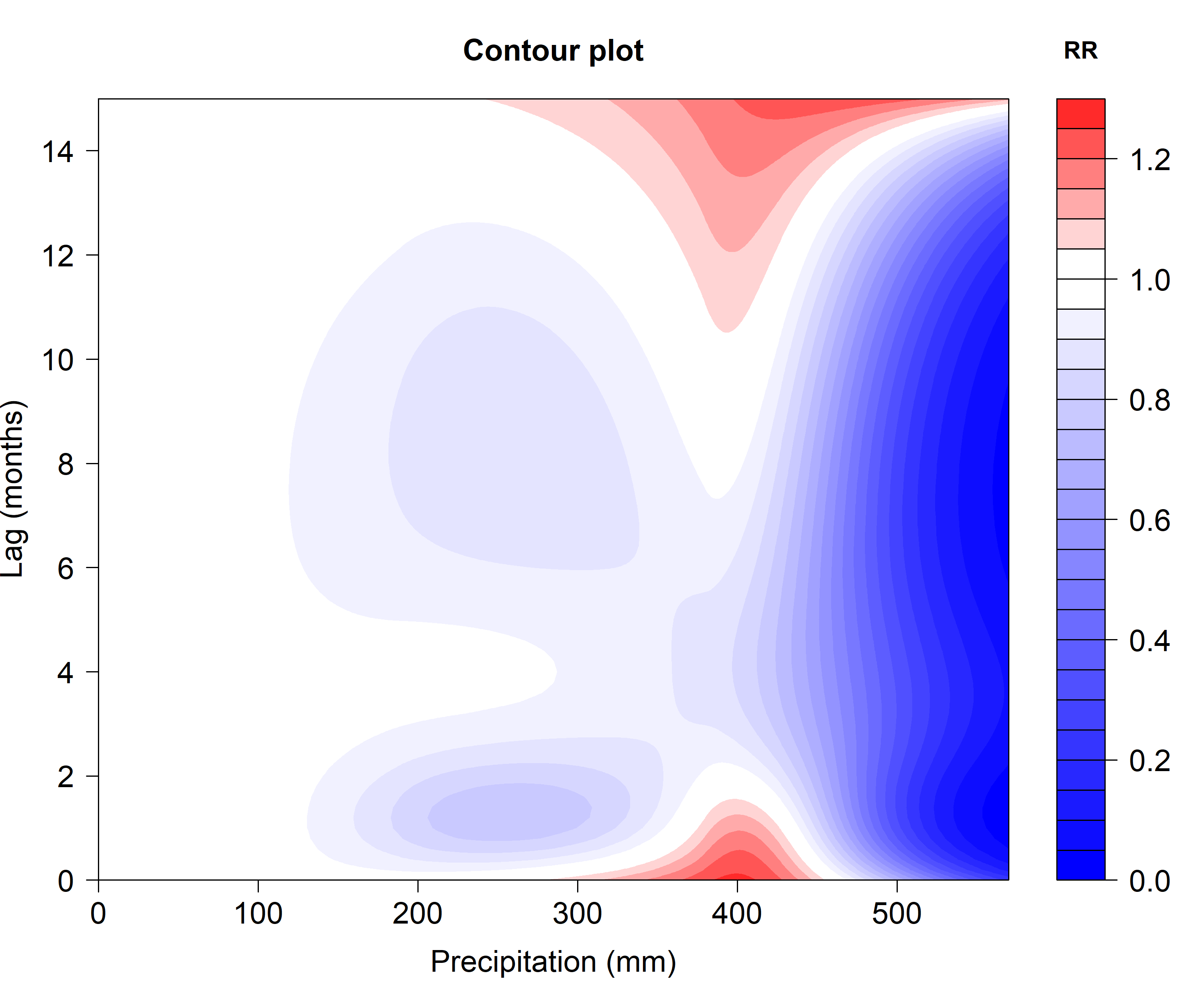

Supplement: Supplementary file 4 — Source Data [file 41467_2020_17987_MOESM4_ESM.zip › FIGURE 6/FIGURE_6F.tiff]

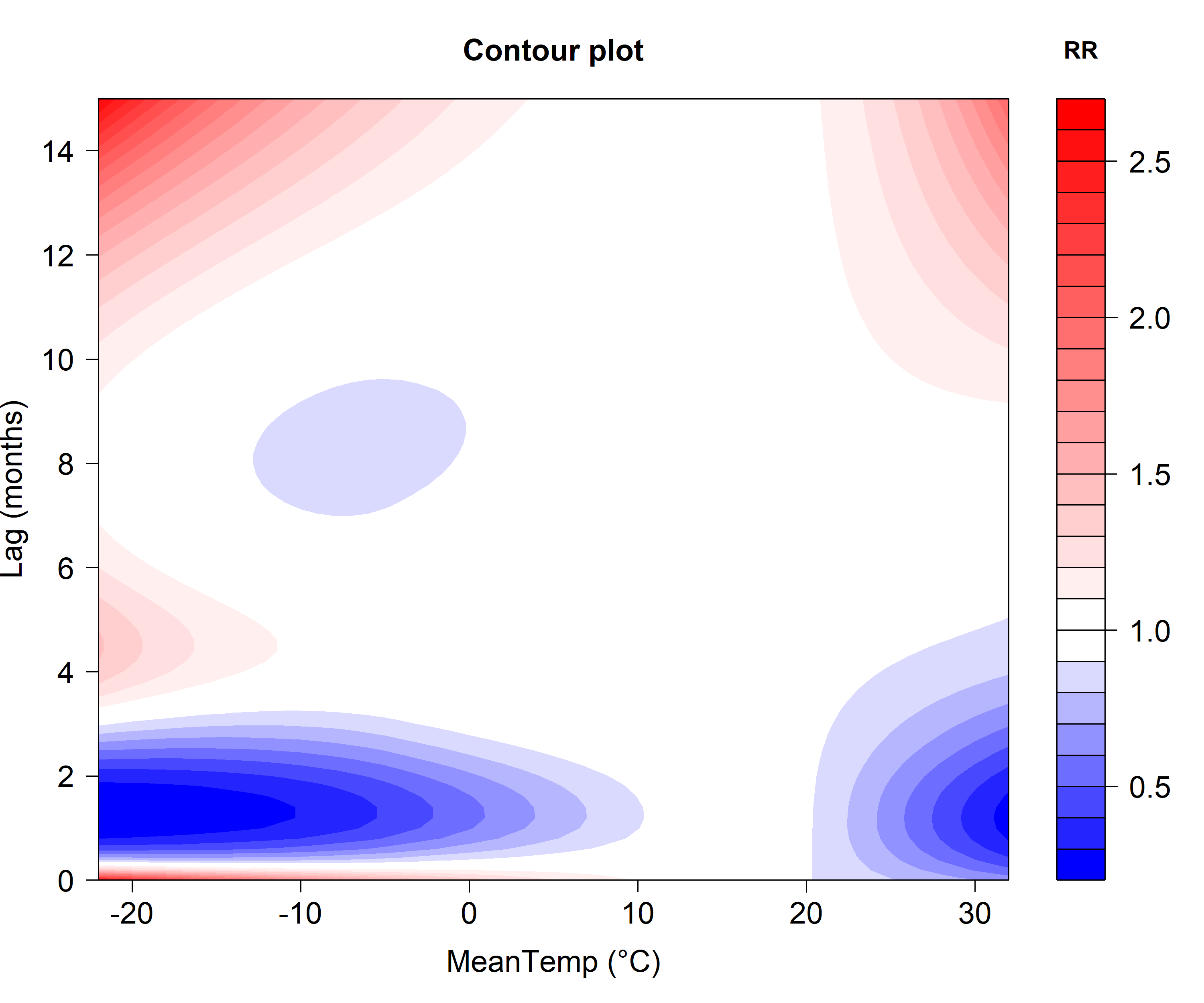

Supplement: Supplementary file 4 — Source Data [file 41467_2020_17987_MOESM4_ESM.zip › FIGURE 6/FIGURE_6G.tiff]

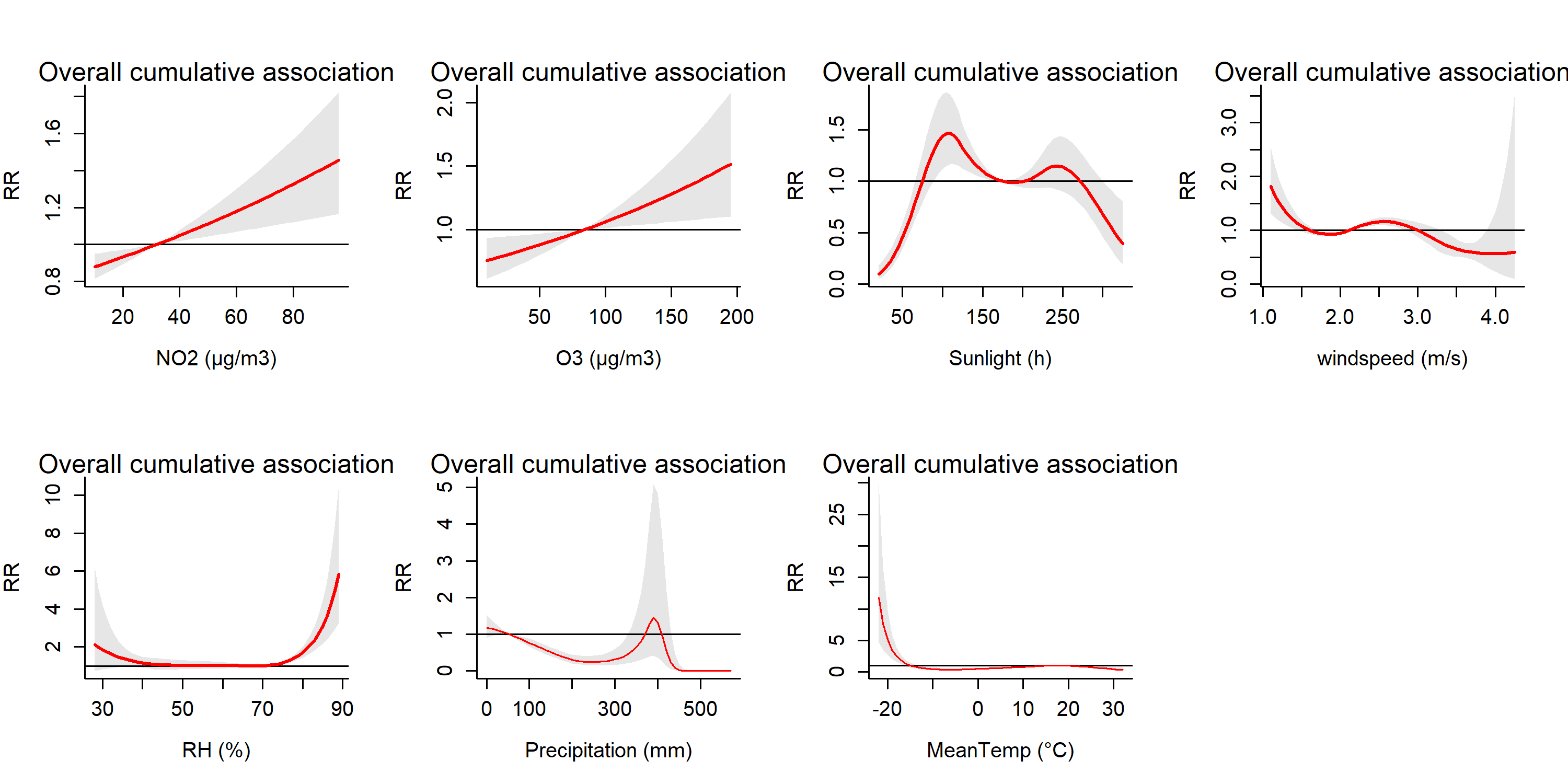

Supplement: Supplementary file 4 — Source Data [file 41467_2020_17987_MOESM4_ESM.zip › FIGURE 7/FIGURE 7.tiff]

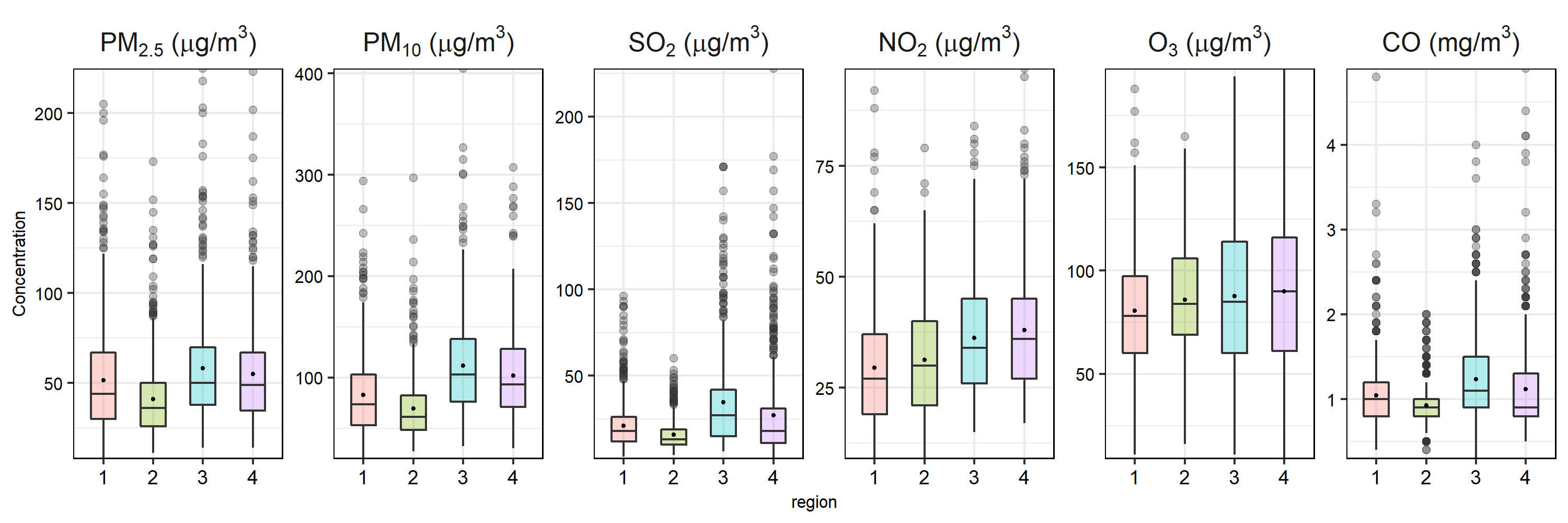

Supplement: Supplementary file 4 — Source Data [file 41467_2020_17987_MOESM4_ESM.zip › SUPPLEMENT 11/SUPPLEMENT 11.tiff]

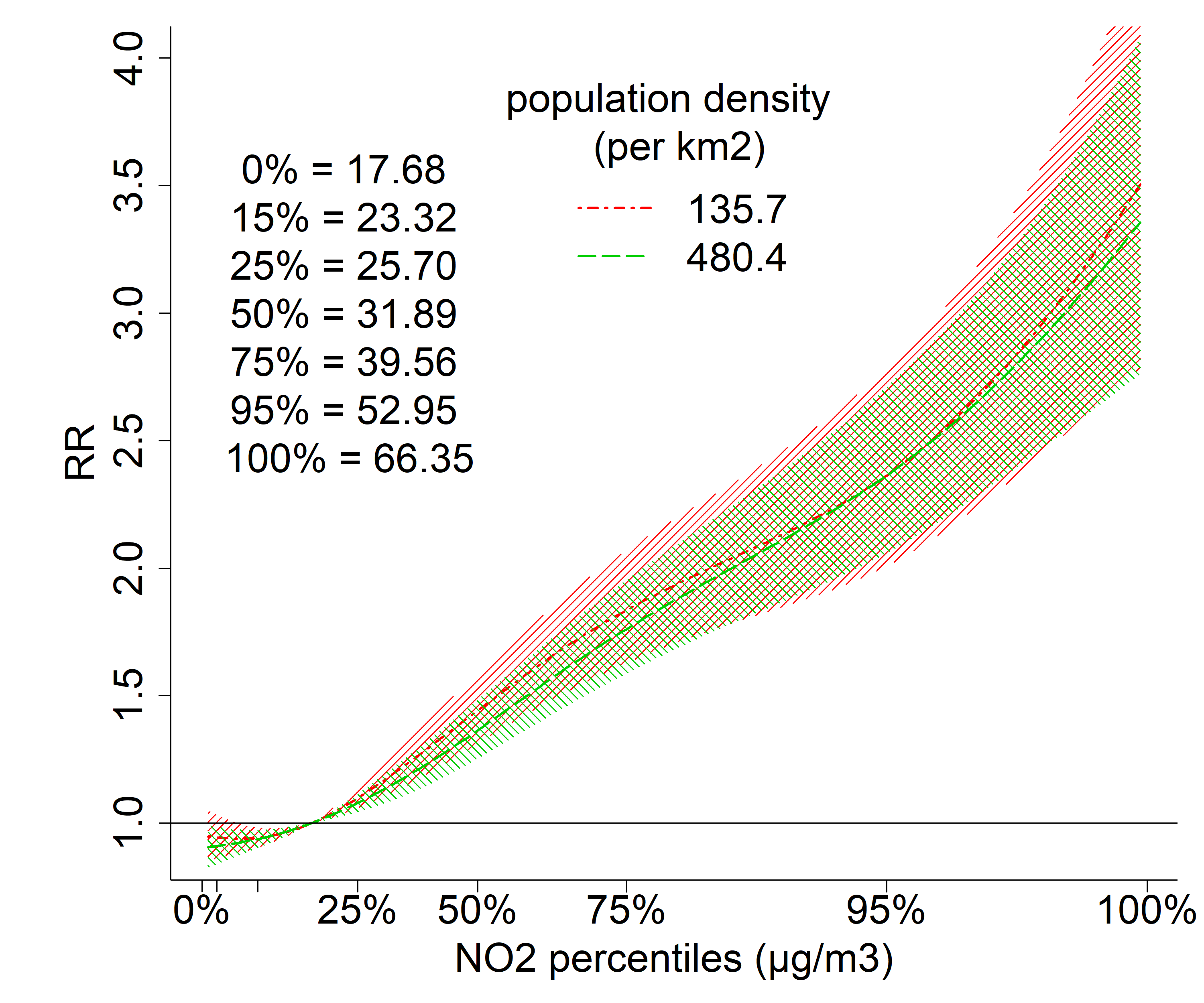

Supplement: Supplementary file 4 — Source Data [file 41467_2020_17987_MOESM4_ESM.zip › SUPPLEMENT 15/SUPPLEMENT_15A.tiff]

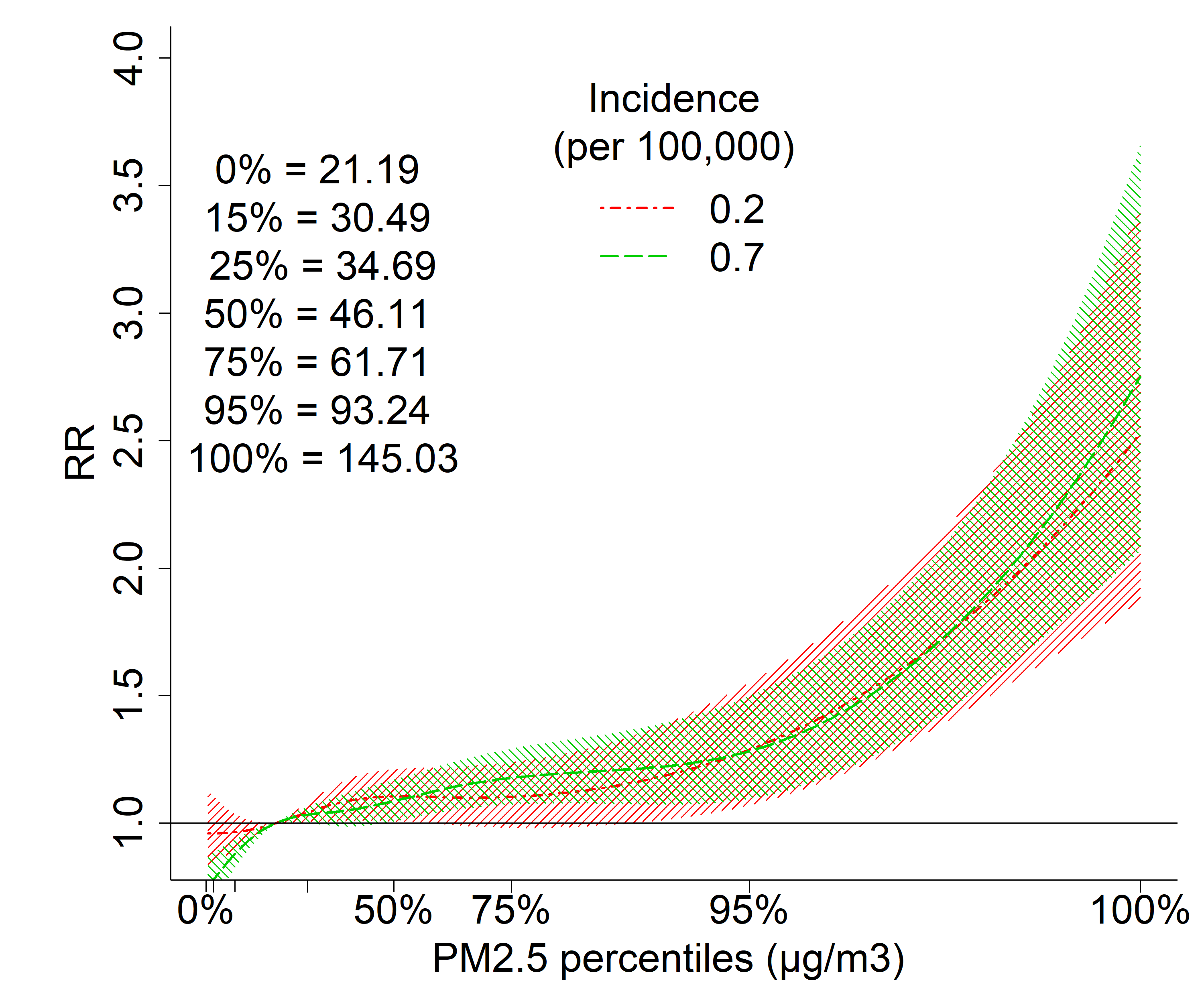

Supplement: Supplementary file 4 — Source Data [file 41467_2020_17987_MOESM4_ESM.zip › SUPPLEMENT 15/SUPPLEMENT_15B.tiff]

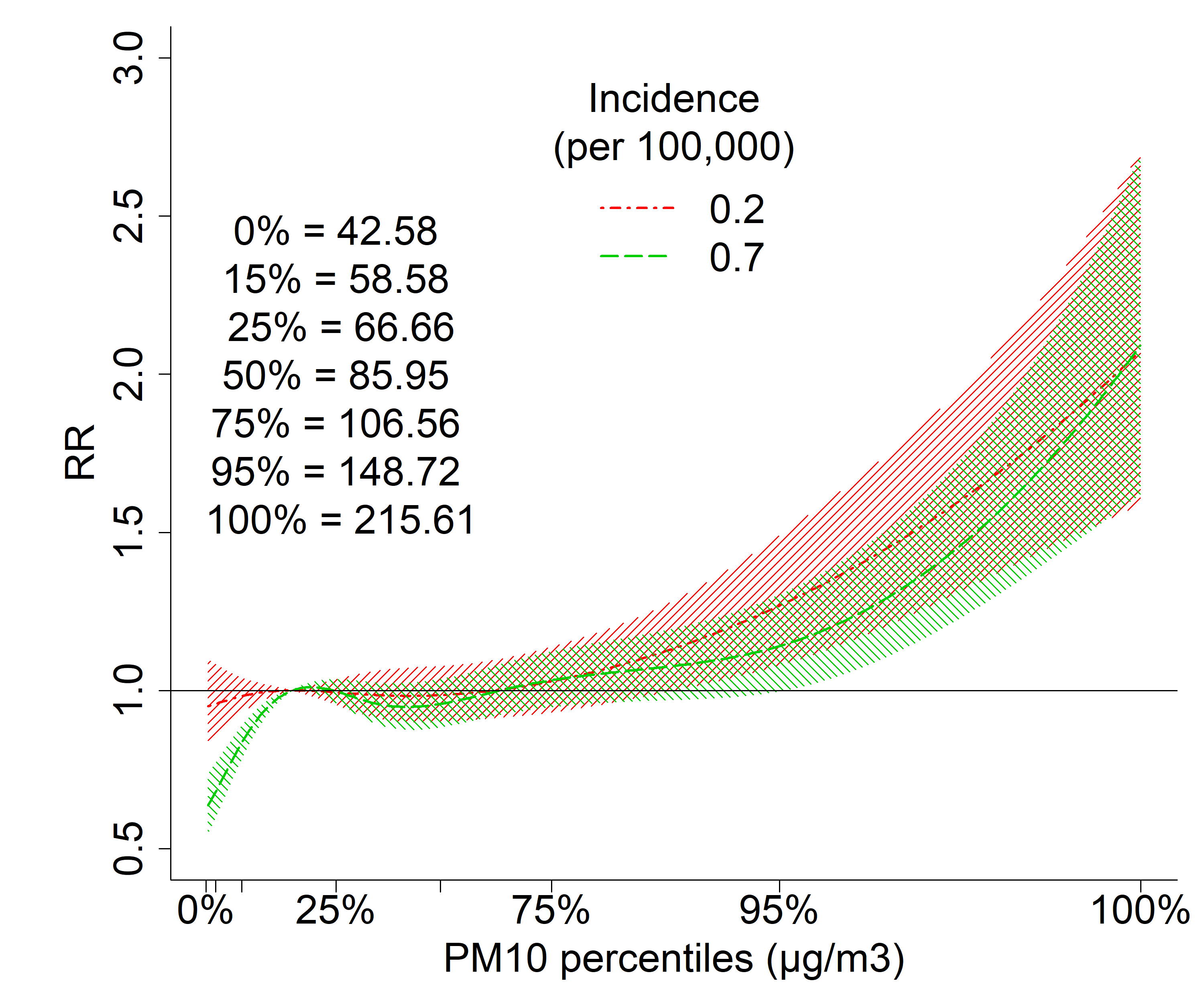

Supplement: Supplementary file 4 — Source Data [file 41467_2020_17987_MOESM4_ESM.zip › SUPPLEMENT 15/SUPPLEMENT_15C.tiff]

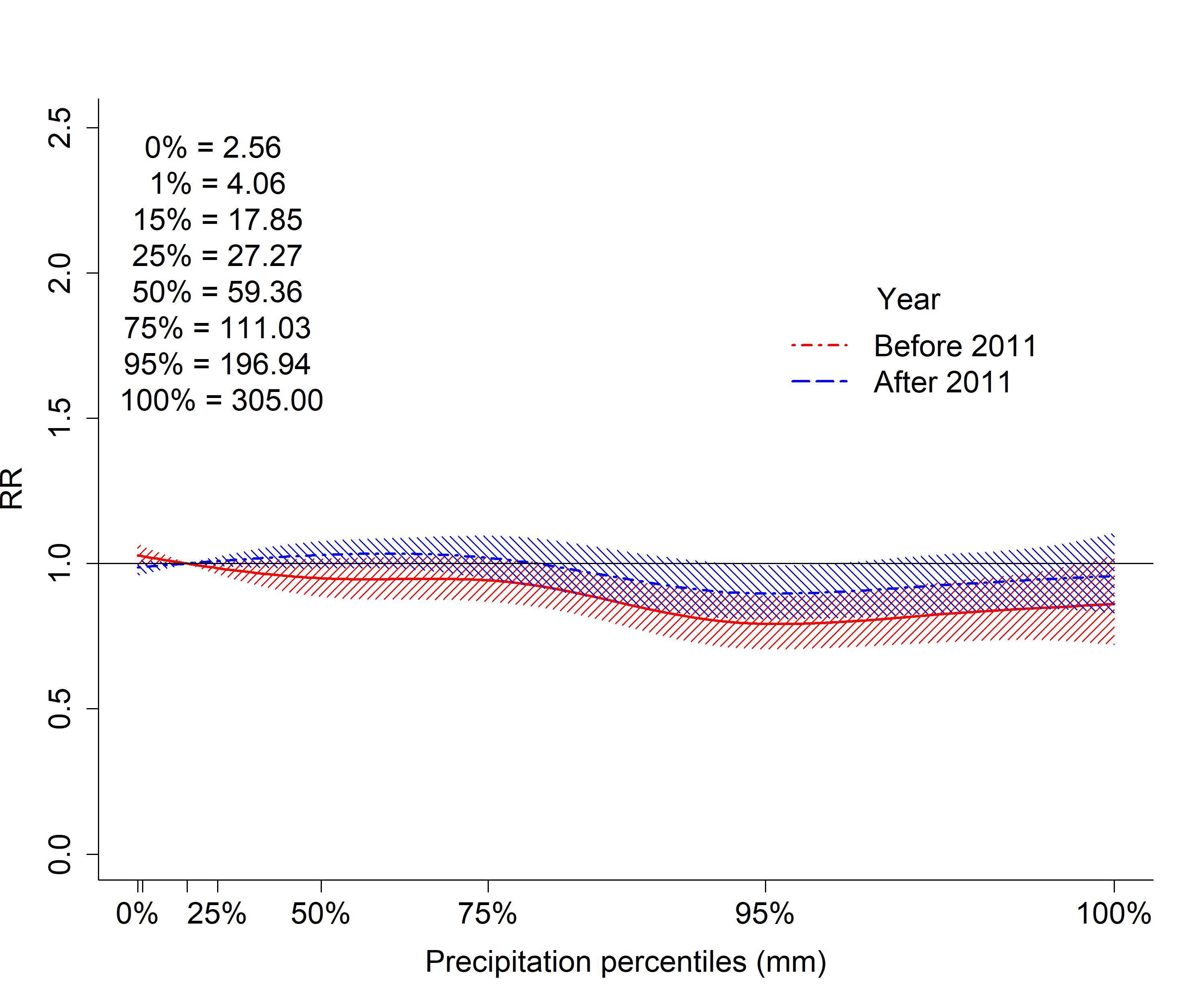

Supplement: Supplementary file 4 — Source Data [file 41467_2020_17987_MOESM4_ESM.zip › SUPPLEMENT 16/SUPPLEMENT_16A.tiff]

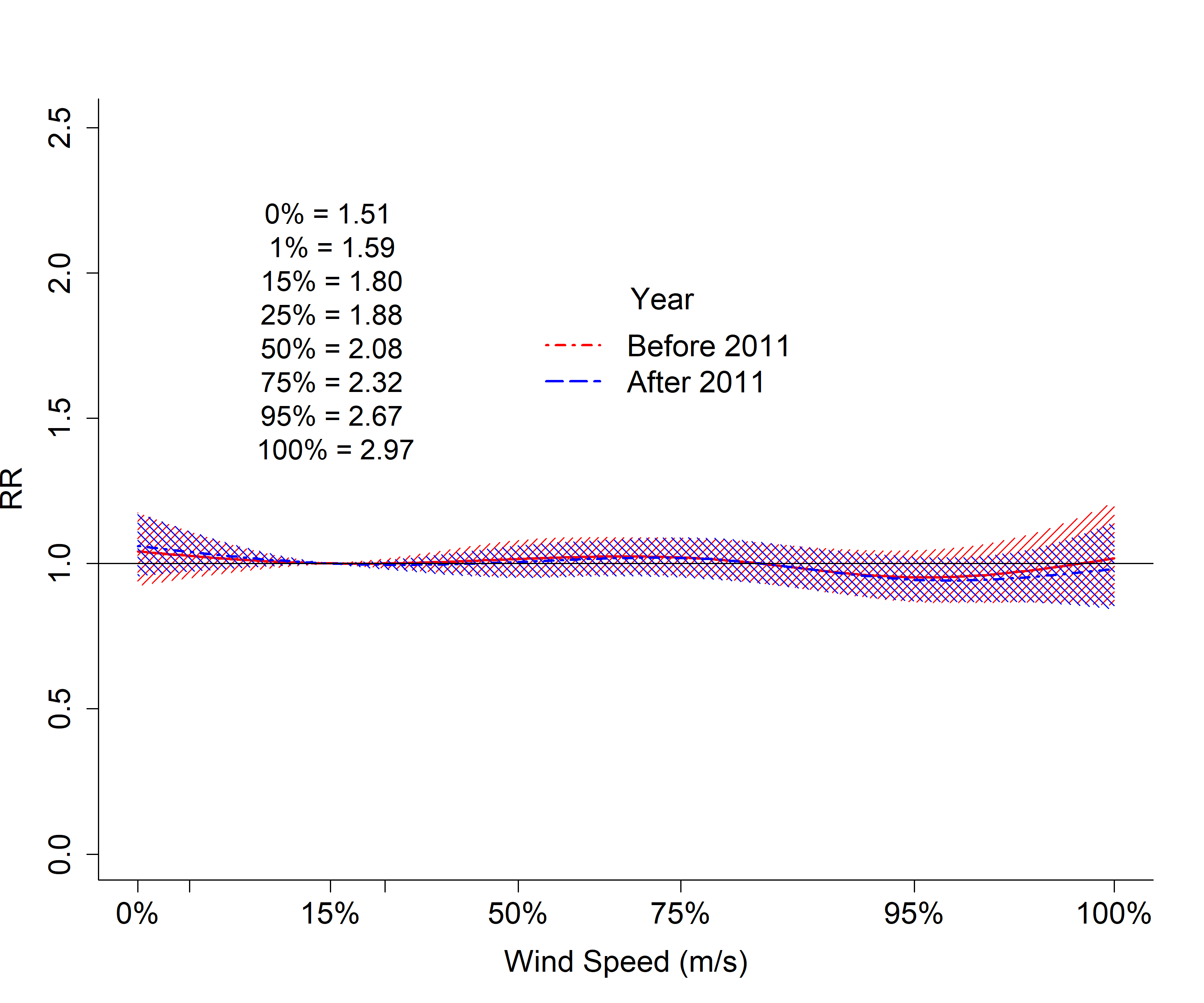

Supplement: Supplementary file 4 — Source Data [file 41467_2020_17987_MOESM4_ESM.zip › SUPPLEMENT 16/SUPPLEMENT_16B.tiff]

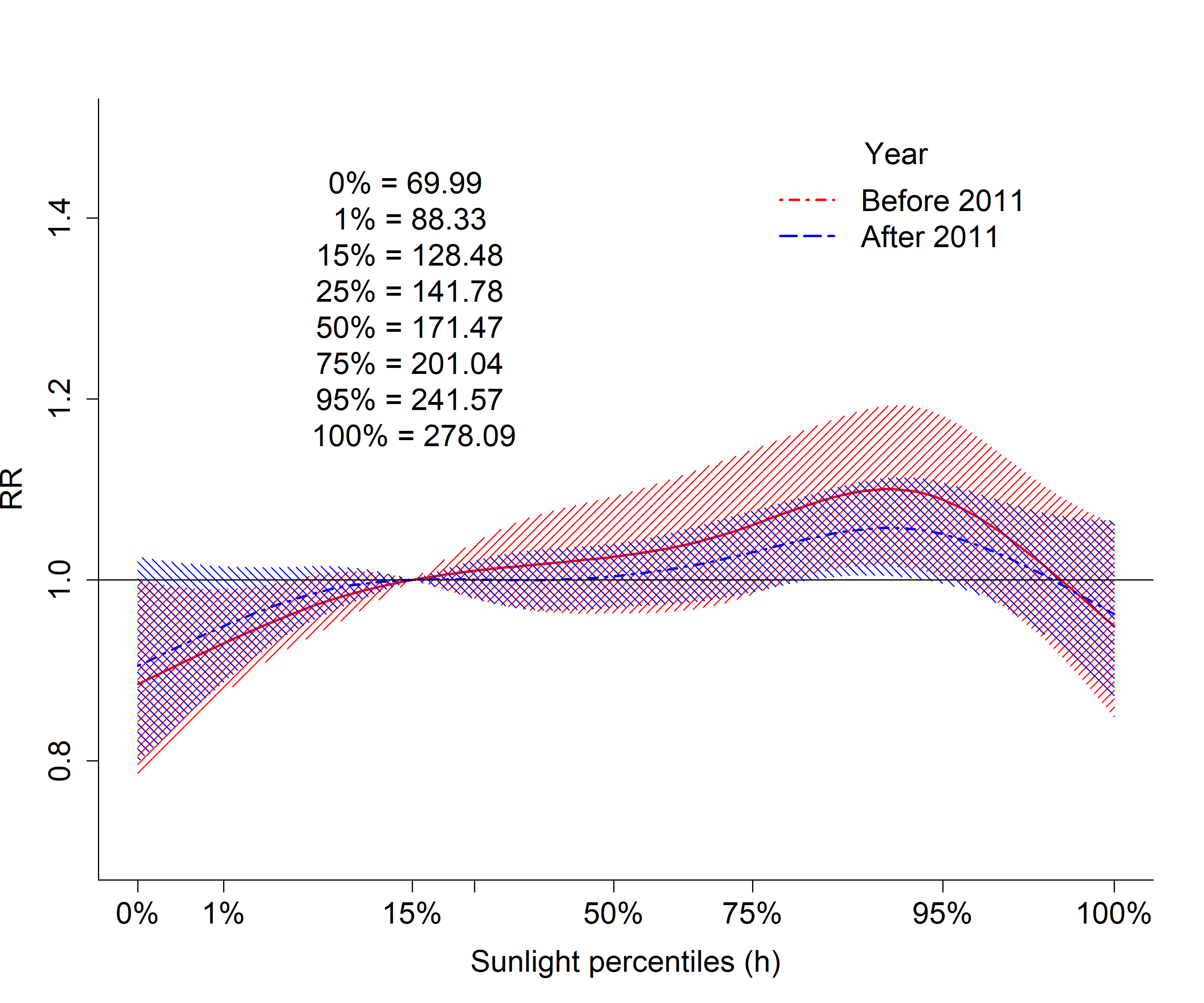

Supplement: Supplementary file 4 — Source Data [file 41467_2020_17987_MOESM4_ESM.zip › SUPPLEMENT 16/SUPPLEMENT_16C.tiff]

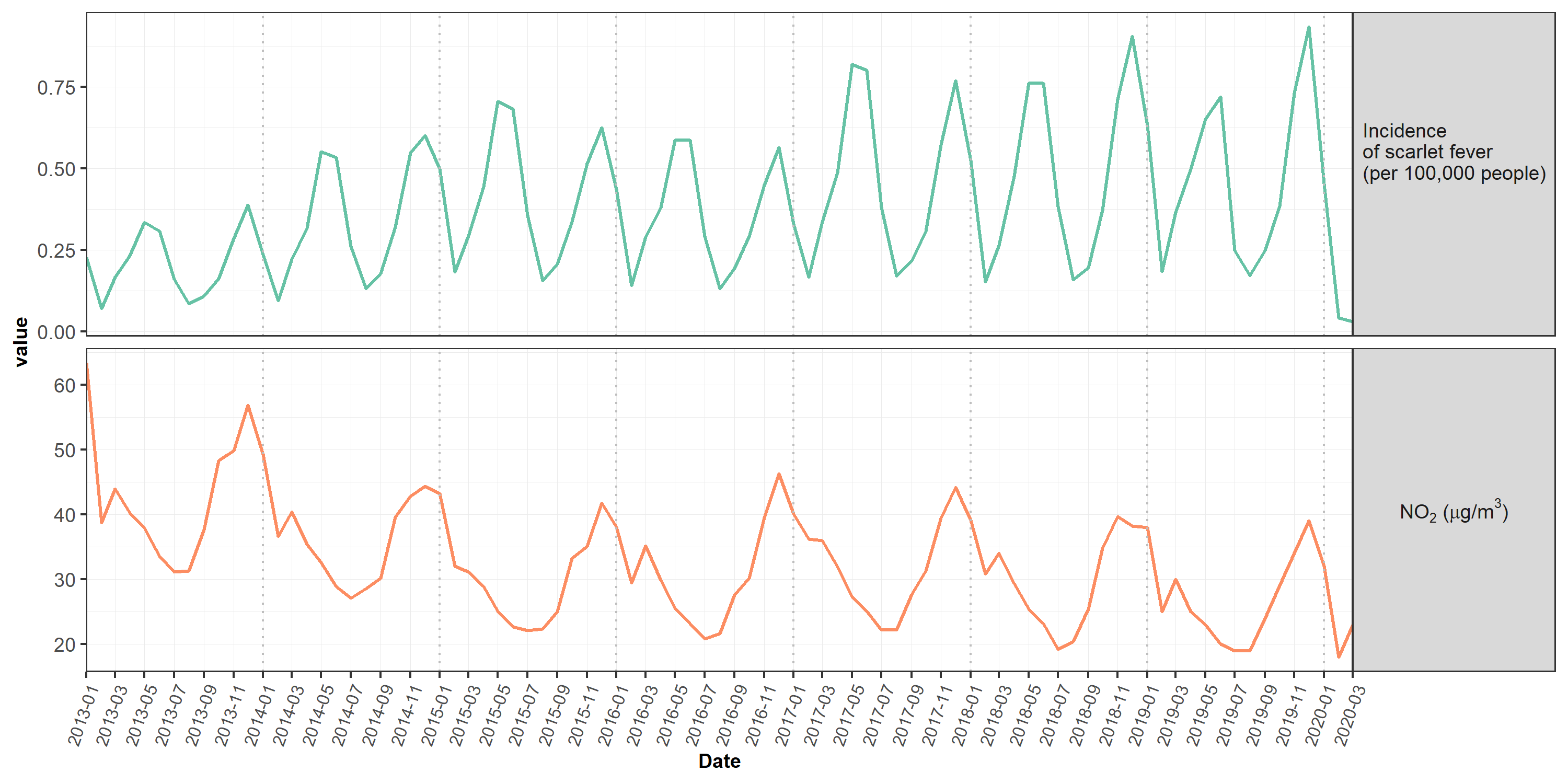

Supplement: Supplementary file 4 — Source Data [file 41467_2020_17987_MOESM4_ESM.zip › SUPPLEMENT 17/SUPPLEMENT_17.tiff]

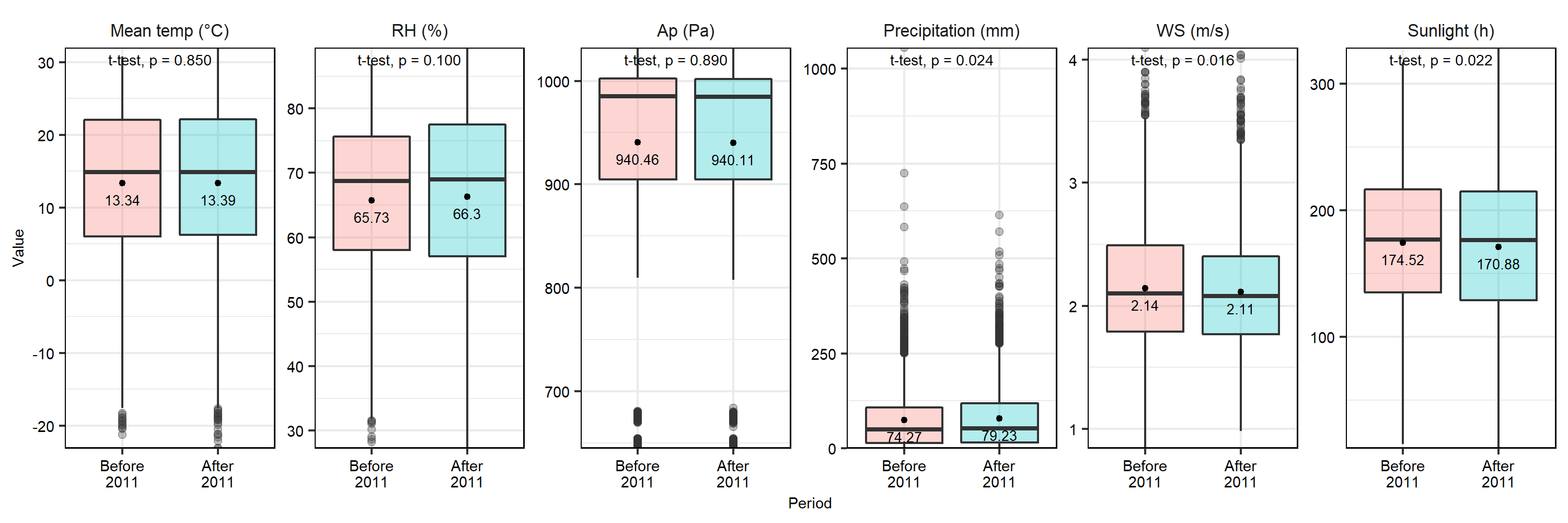

Supplement: Supplementary file 4 — Source Data [file 41467_2020_17987_MOESM4_ESM.zip › SUPPLEMENT 6/SUPPLEMENT_6.tiff]

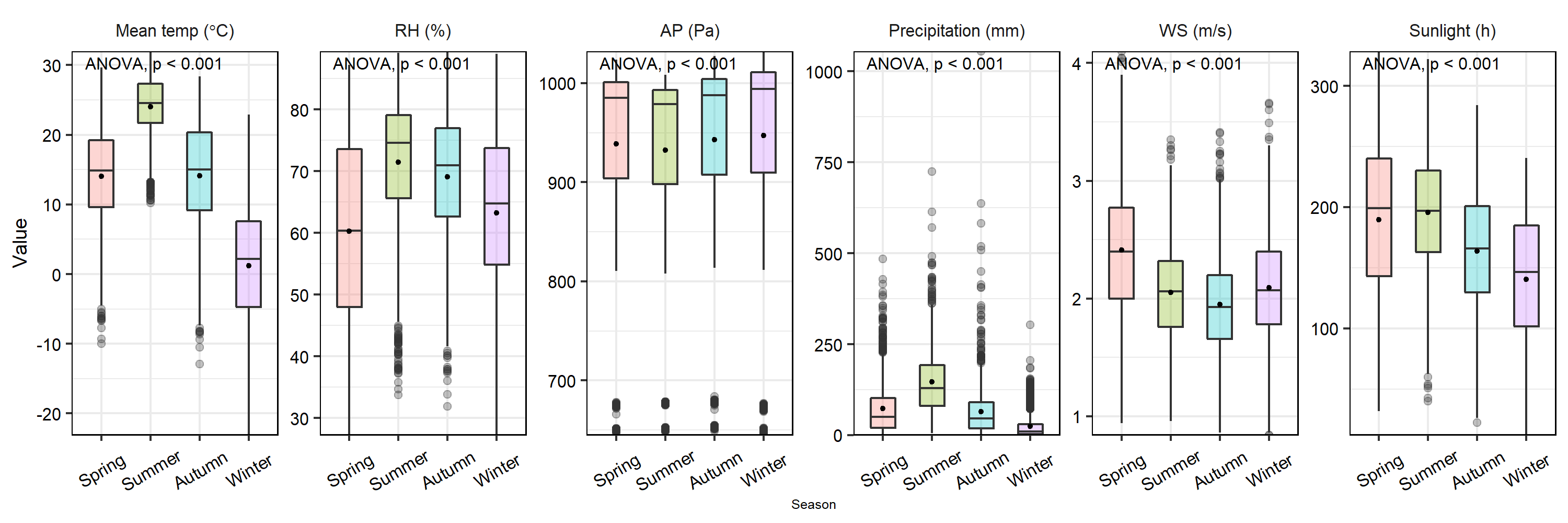

Supplement: Supplementary file 4 — Source Data [file 41467_2020_17987_MOESM4_ESM.zip › SUPPLEMENT 7/SUPPLEMENT_7A.tiff]

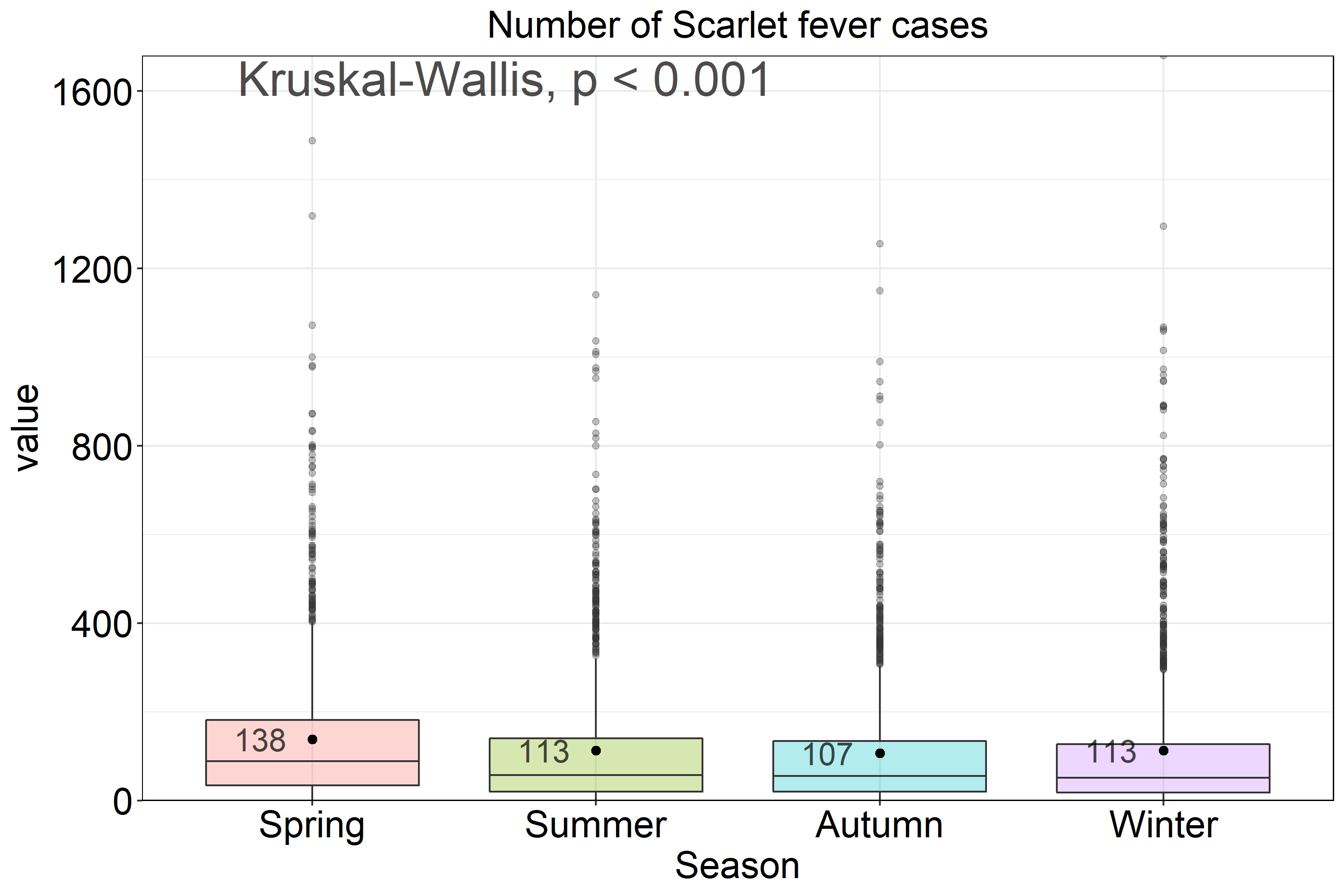

Supplement: Supplementary file 4 — Source Data [file 41467_2020_17987_MOESM4_ESM.zip › SUPPLEMENT 7/SUPPLEMENT_7B.tiff]
